# High-resolution datasets of synthetic human contact network in 13 countries for infectious disease transmission

**DOI:** 10.1016/j.dib.2025.112373

**Published:** 2025-12-09

**Authors:** Zhilu Yuan, Ziyao Luo, Shenyao Lin, Yifang Ma, Yushuang Chen, Mingda Xu, Zhanwei Du, Yuan Bai

**Affiliations:** a State Key Laboratory of Subtropical Building and Urban Science, Shenzhen University, Shenzhen, China; b Research Institute for Smart Cities, School of Architecture and Urban Planning, Shenzhen University, Shenzhen, China; c Faculty of Science, The University of Hong Kong, Hong Kong SAR, China; d WHO Collaborating Centre for Infectious Disease Epidemiology and Control, School of Public Health, Li Ka Shing Faculty of Medicine, The University of Hong Kong, Hong Kong SAR, China; e School of Artificial Intelligence, Optics and Electronics (iOPEN), Northwestern Polytechnical University, Xi'an, China; f The School of Public Health and Emergency Management, Southern University of Science and Technology, Shenzhen, China; g School of Public Health, Sichuan University, Chengdu, Sichuan, China

**Keywords:** Synthetic networks, Social networks, Infectious disease modeling, Epidemiology, Data science, SynthPops

## Abstract

The persistent escalation of global infectious disease threats, exemplified by COVID-19, has resulted in over 30 million deaths worldwide in the past five years. Agent-based models (ABMs) have emerged as powerful tools for simulating disease transmission and assessing intervention efficacy. High-resolution human contact networks are critical components in ABMs, particularly when simulating complex real-world scenarios. However, large-scale, high-quality, and fine-grained publicly accessible human contact network datasets remain scarce. To address this gap, we identified nine key parameters required to generate individual-level synthetic contact networks. Through online searches, we compiled the corresponding parameter values for 13 countries. Using these parameters, we constructed contact networks, each simulating 10,000 individuals. Each individual is characterized by demographic attributes (e.g., age and sex) and multi-layer contact patterns occurring in households, schools, workplaces, and community settings. We validated the generated contact networks by comparing six key socio-demographic metrics with relevant official census data, demonstrating their potential utility in simulating disease transmission dynamics.

Specifications Table

**Table:** 

Subject	Computer Sciences
Specific subject area	Human synthetic contact networks parameterizing ABMs to model infectious disease transmission dynamics.
Type of data	Table (.csv format) Raw
Data collection	Corresponding parameter values for 13 countries were extracted through systematic data collation from official web sources; these were subsequently employed within the SynthPops framework (Python) to generate synthetic contact networks at 10,000-individuals scale.
Data source location	Country: Australia, Canada, France, Germany, Ireland, Israel, Italy, Japan, Spain, Sweden, the Netherlands, the United Kingdom (U.K.), and the United States (U.S.).
Data accessibility	Repository name: Figshare Data identification number: 10.6084/m9.figshare.28466948 Direct URL to data: https://doi.org/10.6084/m9.figshare.28466948 Instructions for accessing these data: Editors and reviewers may access the data via https://figshare.com/s/b0a38ae264b5cdffb3cd
Related research article	None.

## 1 Value of the Data

• These datasets address a critical gap in large-scale, high-resolution, publicly accessible human contact network data. Such high-resolution networks are fundamental for accurately simulating disease transmission dynamics (e.g., COVID-19, Respiratory Syncytial Virus (RSV)) and evaluating intervention efficacy (e.g., vaccination campaigns, workplace and school closures). If other countries can be made available, it will enhance the long-term value of the work.
• The primary beneficiaries of these datasets are interdisciplinary researchers and public health policymakers, including epidemiologists and public health agencies, health economists, immunologists, and social scientists.
• These networks represent all potential transmission pathways, where nodes denote individuals annotated with demographic attributes (e.g., age, household, school, workplace), and edges denote social contacts of various types. Based on such network structures, researchers can employ individual-level stochastic transmission models for simulation [1]: The virus spreads from randomly introduced index cases to susceptible neighbours through active network edges at each time step, governed by predefined, edge-specific transmission probabilities.
• By adjusting the transmission dynamics parameters, the same synthetic contact network datasets can be flexibly applied to simulate the epidemic characteristics of different infectious diseases. When modelling COVID-19 [1], researchers typically assign higher baseline transmission probabilities across all contact types, with particular emphasis on workplace and community mixing layers, to capture adult-driven transmission and the potential for super-spreading events. In contrast, when modelling RSV [2], greater weight is assigned to edges representing contacts within households and preschool settings—the primary environments of infants and young children.
• These datasets enable the assessment of intervention strategies by altering network structures or node states [[2], [3], [4]]. Vaccination is modeled by converting target nodes (e.g., specific age or occupational groups) from susceptible to immune. In COVID-19 simulations, priority is given to older adults and workers; in RSV models, to infants, household members, or mothers to confer household-level immunity. Place closures are represented by temporarily removing corresponding contact edges, such as workplaces and higher education institutions for COVID-19, or preschools and primary schools for RSV, thereby disrupting dominant transmission pathways.

## 2 Background

Rising human migration and population density elevate risks of cross-regional pathogen transmission [5]. Persistent global threats like COVID-19 have caused over 30 million deaths worldwide within five years [6], severely disrupting fundamental societal systems [7]. Mathematical and computational modeling critically supports infectious disease early warning and control, informing public health policies [4,[8], [9], [10]]. Among these, ABMs represent the highest-fidelity epidemiological modeling framework for simulating transmission dynamics and intervention efficacy [1,4]. However, many ABMs struggle to obtain sufficient micro-level data for parameterization, which limits their predictive capability, particularly when inferring individual-level variables is required [11].

Currently available public synthetic contact network datasets exhibit significant limitations [[12], [13], [14]]. First, many datasets are built on an insufficient number of demographic parameters with limited granularity, which constrains the realism and resolution of the generated networks. Second, even when parameters are relatively detailed, the data are often limited to a single country or region, leading to restricted transnational comparability and generalizability of the published synthetic networks. Furthermore, existing datasets often exhibit gaps in the coverage of contact settings—most include only certain types of environments. Consequently, large-scale, high-quality, fine-grained publicly accessible human contact network datasets remain scarce.

## 3 Data Description

**Fig. 1:**
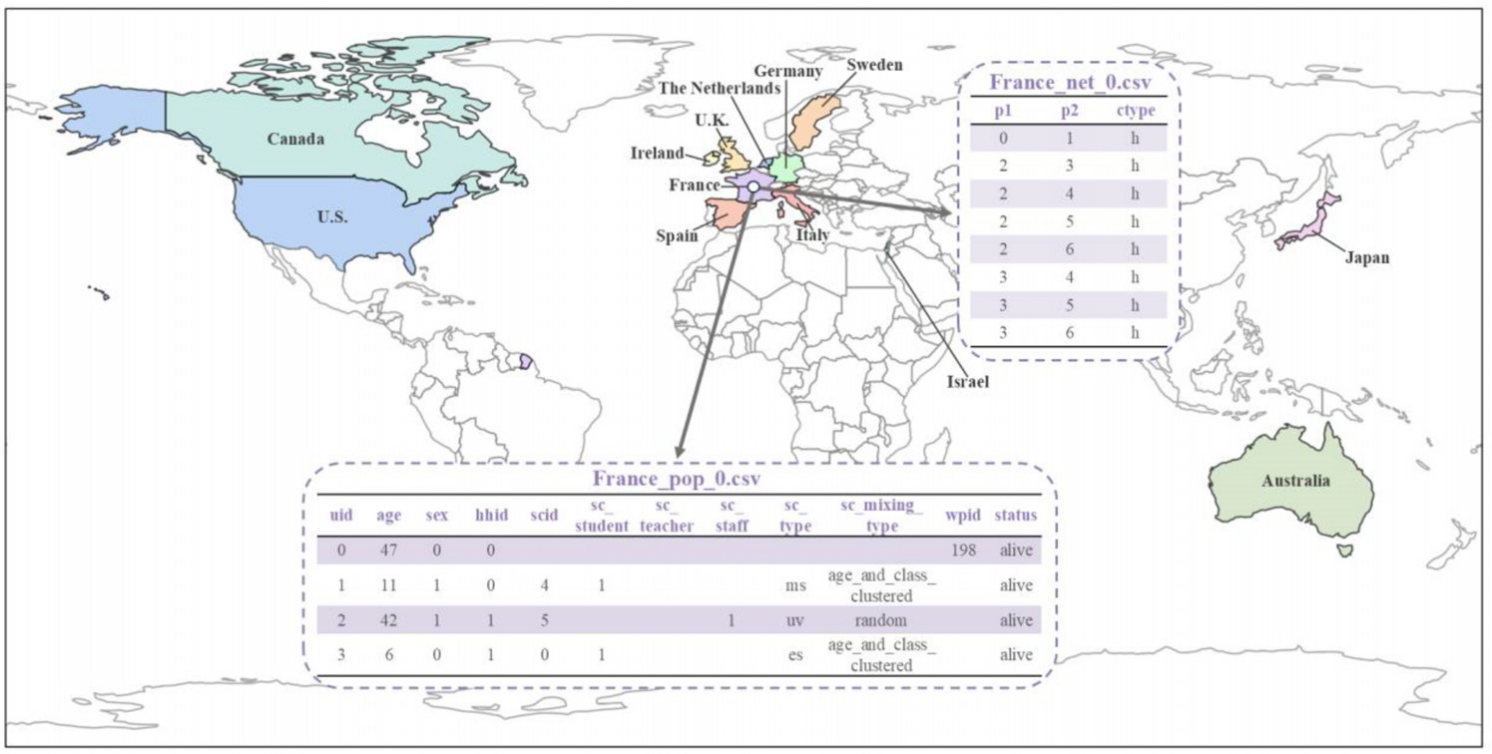
Spatial geographical positions of 13 countries and data overview. The figure illustrates the geospatial positions of the 13 countries included in this study. For France, the figure shows the attribute information and contact types for the first four individuals in the French contact dataset based on the first random simulation. Detailed explanations of these attributes can be found in the following description of CSV files **country_pop_sid.csv** and **country_net_sid.csv**.

**Fig. 2:**
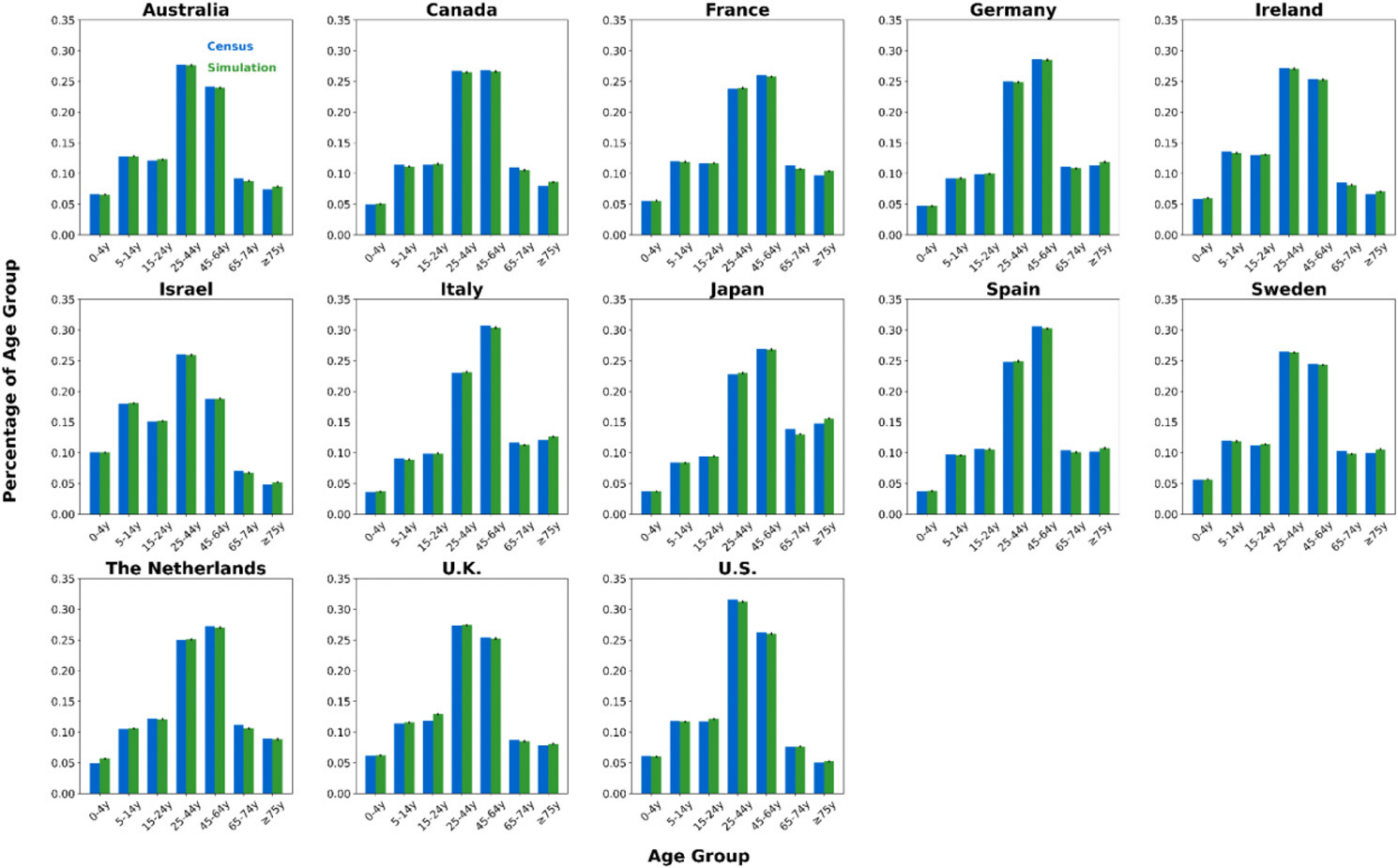
Population age distribution in 13 countries. Age group labels: ``#–#y'' for # to # years, ``≥#y'' for # years and older. For each country, blue bars show official census data, green bars show the mean of 100 simulated networks, with black error lines indicating the 95 % confidence interval bounds.

**Fig. 3:**
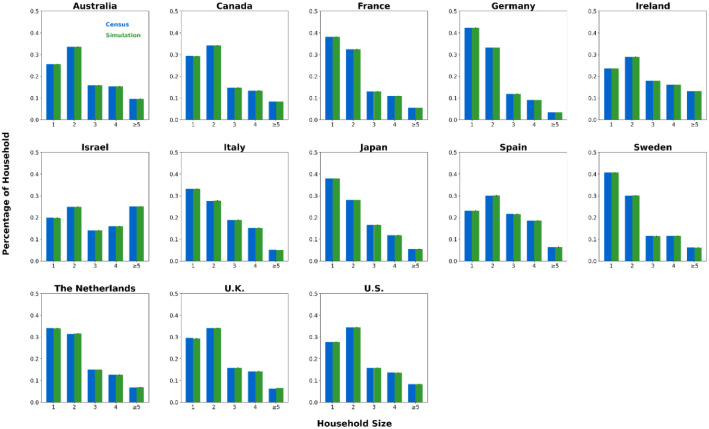
Household size distribution in 13 countries. Size group labels: ``#'' for #-person households, ``≥#'' for households with # or more people. For each country, blue bars show official census data, green bars show the mean of 100 simulated networks, with black error lines indicating the 95 % confidence interval bounds.

**Fig. 4:**
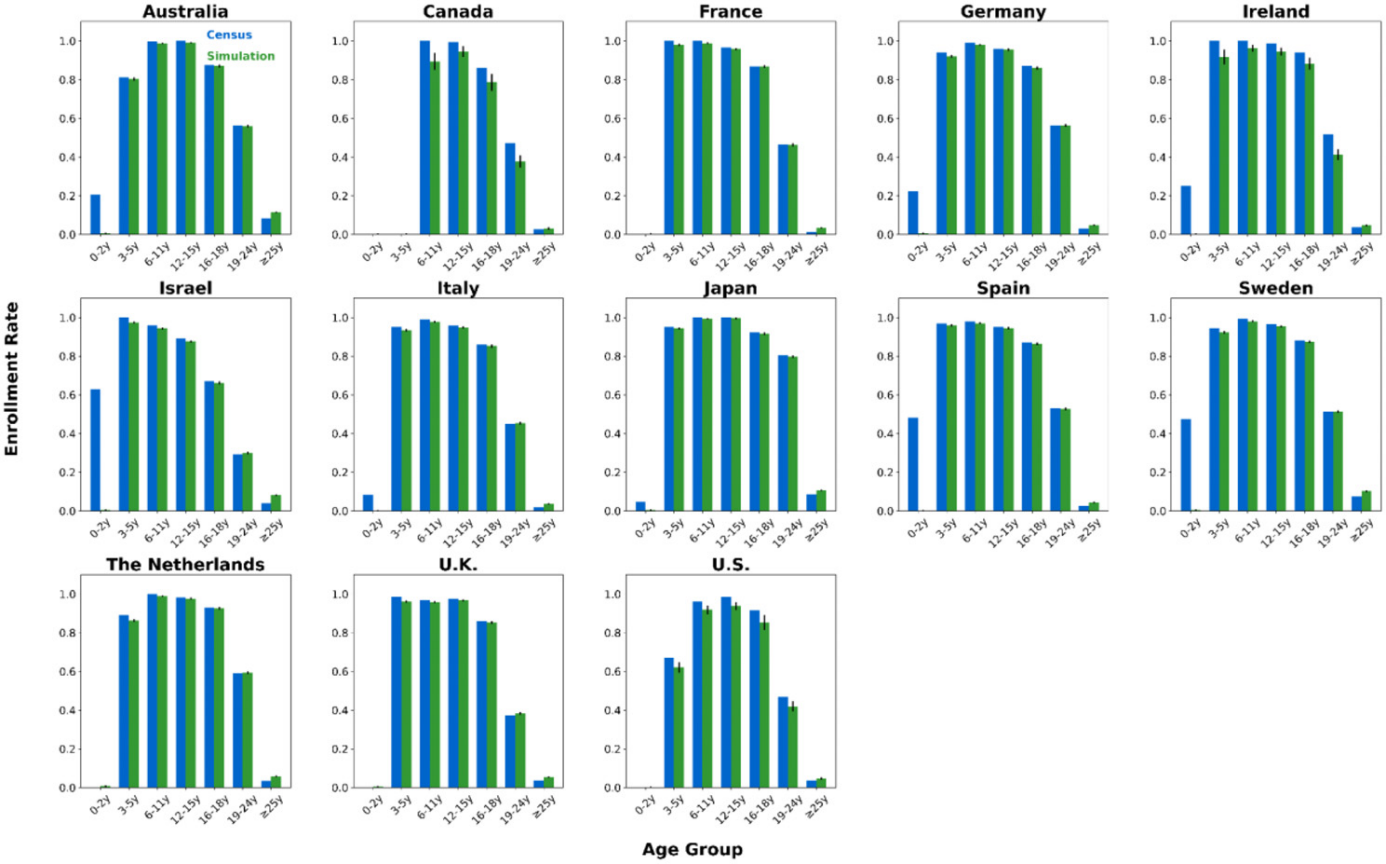
Age-specific school enrollment rates in 13 countries. Age group labels: ``#–#y'' for # to # years, ``≥#y'' for # years and older. For each country, blue bars show official census data, green bars show the mean of 100 simulated networks, with black error lines indicating the 95 % confidence interval bounds.

**Fig. 5:**
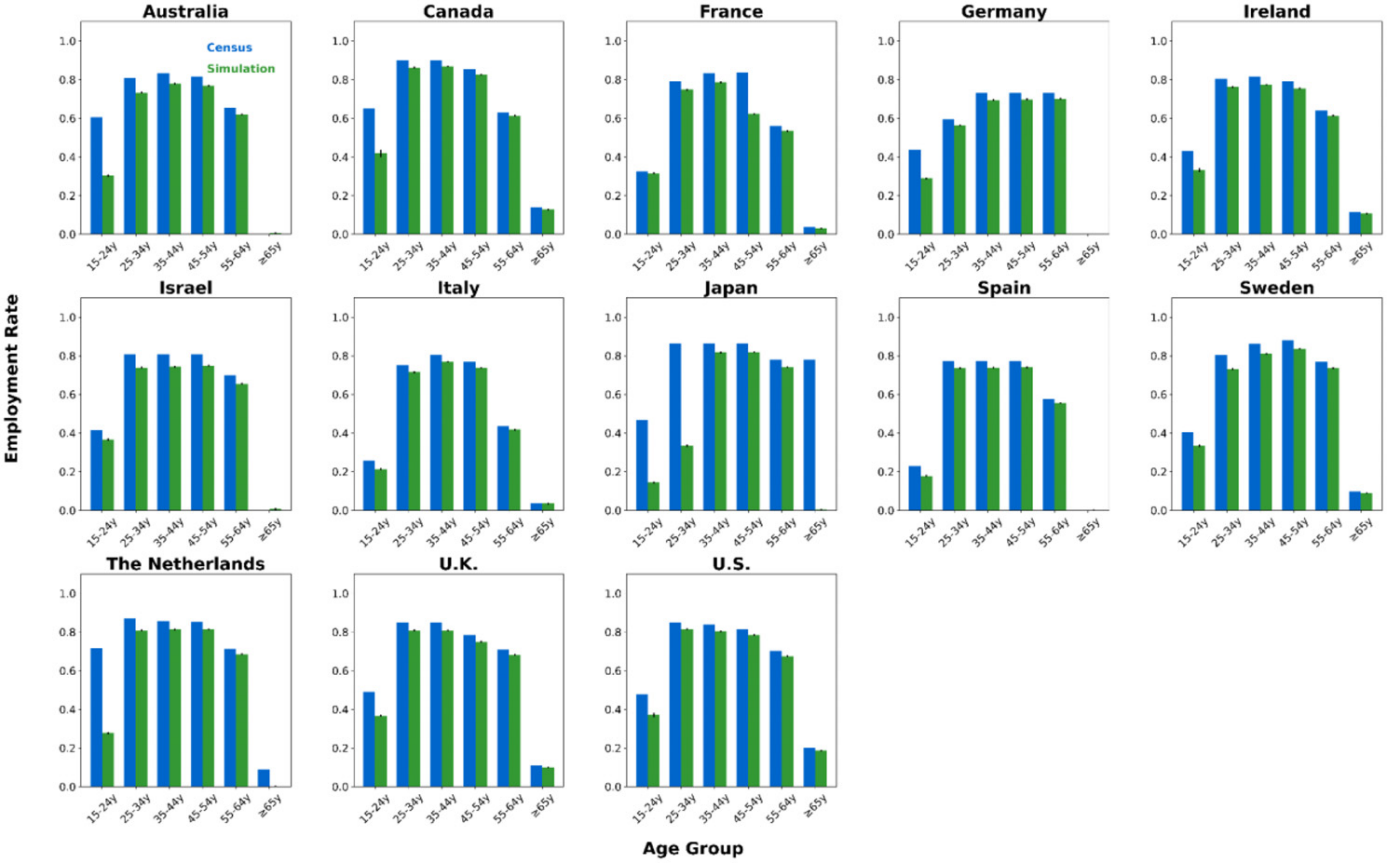
Age-specific employment rates in 13 countries. Age group labels: ``#–#y'' for # to # years, ``≥#y'' for # years and older. For each country, blue bars show official census data, green bars show the mean of 100 simulated networks, with black error lines indicating the 95 % confidence interval bounds.

**Fig. 6:**
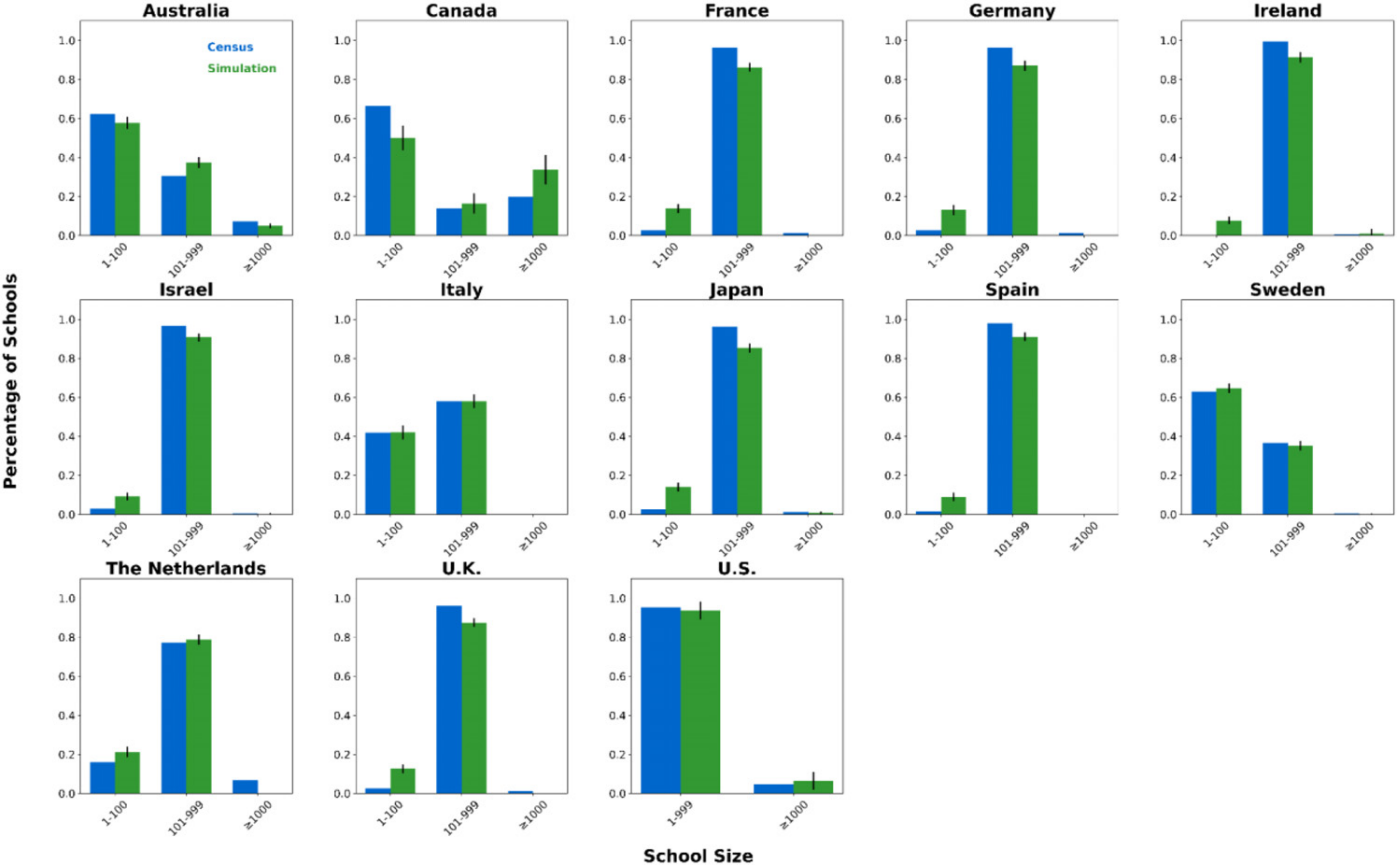
School size distribution in 13 countries. Size group labels: ``#'' for #-person schools, ``≥#'' for schools with # or more people. For each country, blue bars show official census data, green bars show the mean of 100 simulated networks, with black error lines indicating the 95 % confidence interval bounds.

**Fig. 7:**
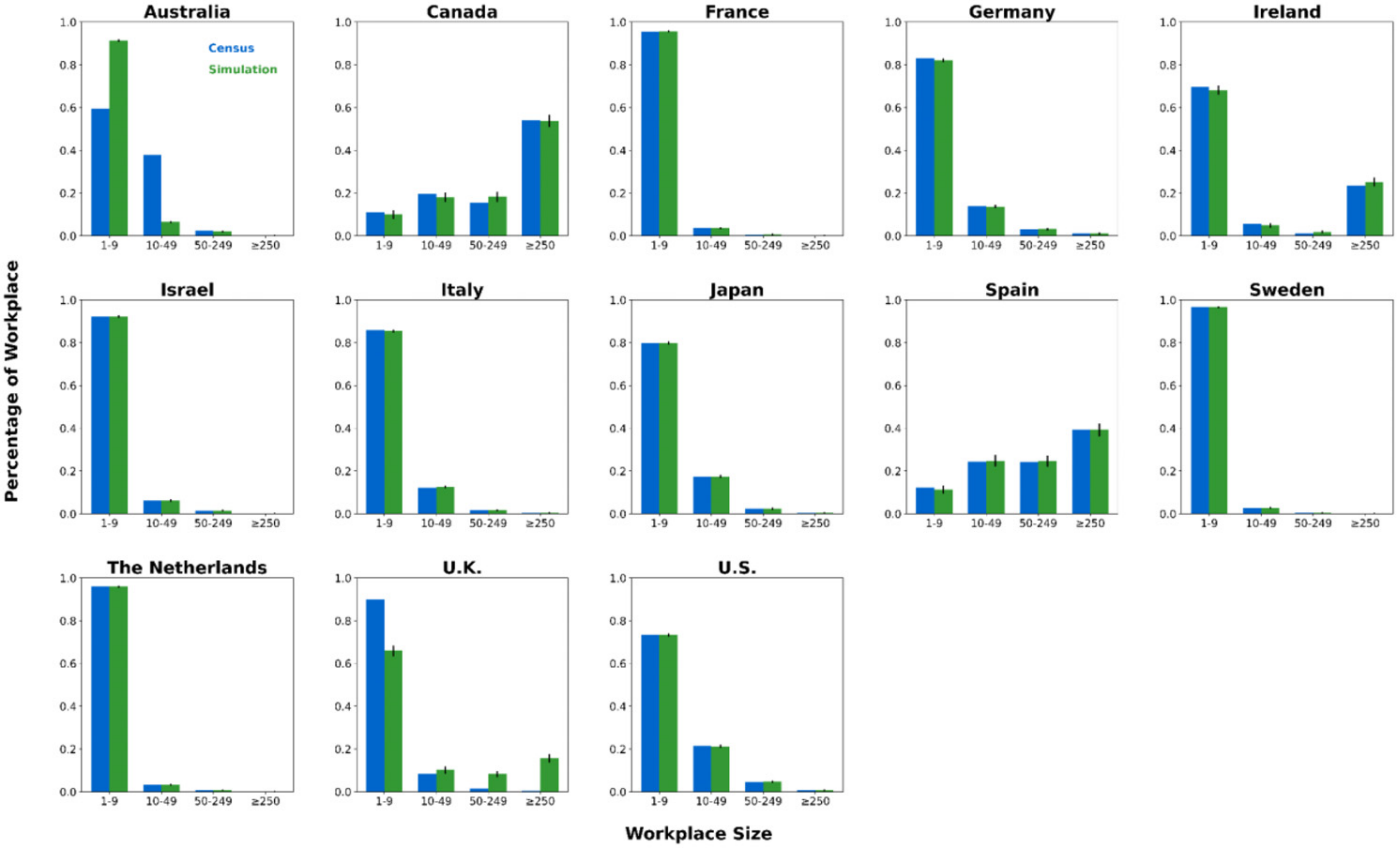
Workplace size distribution in 13 countries. Size group labels: ``#'' for #-person workplaces, ``≥#'' for workplaces with # or more people. For each country, blue bars show official census data, green bars show the mean of 100 simulated networks, with black error lines indicating the 95 % confidence interval bounds.

We generated synthetic contact network datasets by simulating the attributes and behaviors of 10,000 individuals using SynthPops, based on nine parameter values collected from each country. For each country, we generated 100 synthetic contact networks. We saved the individual attributes and contact relationships from the synthetic contact network datasets into two folders, with the specific file format being comma-separated values (CSV) files [15]. The folder **country_pop** is used to store the individual attributes of the contact networks for each country. For example, the file France_pop_0.csv contains the individual attributes for 10,000 individuals in the first contact network synthesized using the 9 parameter values for France (Fig. 1). The folder **country_net** is used to store the link information of the contact networks for each country. For example, the file France_net_0.csv contains the edge list representing the first contact network synthesized for France (Fig. 1). The following demographic characteristics of the contact network datasets are presented in Fig. 2, Fig. 3, Fig. 4, Fig. 5, Fig. 6, Fig. 7, respectively: age distribution, household size distribution, school enrollment rate by age, employment rate by age, school size distribution, and workplace size distribution. A detailed description of the file information contained in the **country_pop** and **country_net** folders is provided below.

• **country:** The contact network datasets include 13 countries, Australia, Canada, France, Germany, Ireland, Israel, Italy, Japan, Spain, Sweden, the Netherlands, U.K., and U.S.. The spatial geographical positions of these countries are illustrated in Fig. 1.
• **sid:** It represents the random simulation index number for generating contact networks with SynthPops in each country, its value includes 0, 1, 2, …, 99.

**country_pop_sid.csv:** Each row in this file records the attributes of individuals in a synthetic network dataset, including 12 attributes. Detailed descriptions of these attributes are provided in Table 1. For the **age** parameter, newborns are assigned ages ranging from 0.1 to 1, with intervals of 0.1 determined by a uniform distribution. For the school contact mixing type attribute, **sc_mixing_type**, the default mixing mode is set to **age_clustered**.

**Table 1:** Parameters in **country_pop_sid.csv.**

Parameter	Description	Value
uid	Individual code	0, 1, 2, …, 9999
age	Age	0, 1, 2, …, 100
sex	Sex	0 for female, 1 for male
hhid	Household code	0, 1, 2, …
scid	School code	0, 1, 2, …
sc_student	Be a student or not	1 for yes, else for no
sc_teacher	Be a teacher or not	1 for yes, else for no
sc_staff	Be a non-faculty staff or not	1 for yes, else for no
sc_type	School type	pk: pre-kindergarten es: elementary school ms: middle school hs: high school uv: university
sc_mixing_type	The mixing type of the school	random: random graphs for each school age_clustered: students mostly mix within the age/grade age_and_grade_clustered: students are grouped into classes with their teachers
wpid	Workplace code	0, 1, 2, …
status	Individual survival state	alive, dead, newborn, new mother

**country_net_sid.csv:** Each line in this file records a contact event, represented by three columns that include the IDs of the two nodes involved in the contact and the social setting where the contact occurred. Detailed descriptions of these parameters are provided in Table 2. For example, in the file **France_net_0.csv**, a record such as (0, 1, h) denotes a contact between node 0 and node 1 in the household setting **(**Fig. 1**)**. The numbers 0 and 1 correspond to the uid of individuals 0 and 1 in the file **France_pop_0.csv (**Fig. 1**).**

**Table 2:** Parameters in **country_net_sid.csv.**

Parameter	Description	Value
p1	Source node	0, 1, 2, …, 9999
p2	Target node	0, 1, 2, …, 9999
ctype	Contact type	h: household contact s: school contact w: work contact c: community contact

## 4 Experimental Design, Materials and Methods

### 4.1 Input data collection

The nine key parameters required for generating synthetic contact networks using SynthPops are listed below. Based on comprehensive criteria encompassing demographic scale, economic profile, data completeness, and epidemiological severity, we rigorously selected 13 countries for analysis: Australia, Canada, France, Germany, Ireland, Israel, Italy, Japan, Spain, Sweden, the Netherlands, U.K., and U.S. [2,16]. We garnered the corresponding parameter values for each country through systematic data collation from official web sources. Detailed information on the input data and its sources can be found in Supplementary Tables S1–S10. The complete raw input data can be accessed directly at: https://github.com/DEEMOALICE/SynConNet (Folder “Input Data”).

• **Population age distribution**: The proportion of people in different age groups within the total population, reflecting the characteristics of the population age structure.
• **Household size distribution**: The proportion of households of different sizes (e.g., 1-people, 2-people, 3-people households) within the total number of households, used to simulate household composition.
• **Household head age distribution by family size**: The age distribution of household heads in households of varying sizes is used to simulate the age of the reference individuals in different household compositions.
• **School size distribution**: The proportion of schools grouped by size (number of students) is used to model the capacity of educational institutions.
• **Workplace size distribution**: The proportion of workplaces of different sizes (number of employees) within the total number of workplaces, used to simulate work environments.
• **Employment rate in workplaces**: The proportion of people in specific age groups who are employed, used to simulate workforce allocation.
• **Enrollment rate in schools**: The proportion of people in specific age groups who are enrolled in school, used to allocate students, teachers, and non-teaching staff.
• **Birth rate**: The percentage of new population additions relative to the total population within a specific period, used to simulate population growth in a specific region.
• **Death rate**: The percentage of population decrease resulting from deaths relative to the total population within a specific period, used to simulate population reduction in a specific region.

We conducted Google searches using keyword combinations in the “[Country] + [keyword]” format, as detailed in Table 3. We examined the search results sequentially according to their page rankings. Specifically, if a parameter meeting our criteria was found in the third entry, we did not proceed to the fourth and terminated the search. Otherwise, we continued reviewing results up to the tenth page of Google search results, using the completion of the tenth page as the exit condition. We prioritized top-ranked entries—typically those from official institutions—for their higher credibility and accuracy.

**Table 3:** Keywords for search strategy.

Parameters	Keywords
Population age distribution	population age distribution, age structure, age composition, age-sex pyramid, population pyramid, demographic structure by age and sex, population by age group, population by five-year age group, demographic breakdown by age, age dependency ratio, median age of population
Household size distribution	household size distribution, number of persons per household, average household size, private households by size, household composition, household occupancy, household members per dwelling
Household head age distribution by family size	age of household head by family size, age of householder by household size, household composition by age and size, households by age and sex of reference person and by size, family households by age of reference person, demographic characteristics of household head
School size distribution	school size distribution, number of students per school, average enrollment per school, school population size, education institution size distribution, number of schools by enrollment size, number of educational establishments by size
Workplace size distribution	workplace size distribution, establishment size distribution, firm size distribution, enterprise size, number of employees by establishment size, business establishments by employment size, company size classes, business counts by size class
Employment rate in workplaces	employment rate, job density per establishment, employment-to-population ratio, employee rate, labor force participation rate, employment intensity by firm size, proportion of employed persons by workplace
Enrollment rate in schools	school enrollment rate, gross enrollment ratio, net enrollment rate, participation rate in education, education participation rate, enrollment ratio by education level, student enrollment percentage
Birth & Death rate	crude birth rate, crude death rate, mortality rate, fertility rate, natural increase rate, demographic indicators, life expectancy at birth, vital statistics, population growth rate, death-to-birth ratio

*Note:* If content on a website is provided exclusively in the native language, all searches should be conducted in that same language.

After an initial screening to assess the completeness of parameters and their conformity with our collection criteria, we verified the publishing institution by examining the URL domain (e.g., .gov, .org, .int) and the host information. If the source met these institutional criteria, it was recorded; otherwise, we continued to the next entry until reaching the exit condition. If no suitable entry was identified by the exit point, we returned to the first parameter entry that satisfied the basic criteria (even if it was not from an official source, such as data from commercial platforms or data visualization websites) and recorded it. Our key inclusion criteria comprised: (1) data covering the period from 2010 to 2024; (2) availability of distributions categorized by age or size groups. If the data originated from a national statistical website (thus limited to data pertaining to that country), we conducted additional internal searches using keywords related to other parameters of the same country. For data obtained from international organizational databases or from commercial data platforms/data visualization websites, we recorded the source for subsequent searches concerning other countries.

For parameters that could not be identified through initial country-specific searches or national statistical websites (i.e., upon reaching the exit condition), we adjusted our search strategy accordingly. First, we conducted a broader Google search using the core keywords without the country name. If this approach did not yield relevant results, we consulted our pre-compiled international databases, performing searches within them using the “[Country] + [keyword]” format. (At this point, the process returned to the first step and was repeated as necessary.) The data obtained through this stage were typically derived from international organizations or from commercial data platforms/data visualization websites. If a specific parameter remained unavailable after all the above steps had been executed and the exit conditions were reached in each case, we substituted the data with information from a neighboring country that shared similar geographical, cultural, and socioeconomic characteristics.

After collecting all parameters, we harmonized their reference years on a country-specific basis. If the time span between parameters exceeded ten years, we used the earliest year as the benchmark and adjusted the other years accordingly. This process ensured that all data for a given country fell within a ten-year window (e.g., limiting other parameters to ≤2022 when Irish household data was only available in 2012). Finally, we verified the distribution data by confirming that the sums across all categories equaled 1, thereby ensuring internal consistency.

### 4.2 Contact networks generation

These networks were generated using the Python third-party library SynthPops [17]. The runtime environment requires Python 3.6 or higher (64-bit), we recommend installing the latest version of SynthPops (1.10.x). We employed the global social contact matrix constructed by Prem et al. (2017) [18], a dataset covering 152 countries worldwide—including all 13 countries examined in this research.

In generating contact networks, the household, school, and workplace layers are modeled independently, yet they follow the same computational logic. First, individuals and their attributes are generated based on input statistical parameters to conform to specified statistical distributions. Second, the contact matrix is used to calculate the probability of contact between individuals. Finally, an undirected human contact network (i.e., edges)—is generated based on these probabilities. Although the layers in the network are independent, transmission rates in the infectious disease model can be calibrated using mortality and morbidity data, allowing for a more accurate assessment of intervention effects across different settings [19].

In addition, we randomly assigned attributes such as being **alive, dead, newborn**, and **new mother** to individuals based on the birth and death rates of respective countries. This enables the model to support studies on infectious diseases such as RSV. The following three subsections summarize how to use SynthPops to generate contact networks for different social settings. Python codes and detailed tutorials for generating and validating these networks are available at https://github.com/DEEMOALICE/SynConNet.

### 4.3 Households contact layer

The model generates household members for specific populations by integrating age distribution, household size, and the age of household heads. Specifically, the algorithm employs the household size distribution parameters from input statistical data to determine the sizes of households. Thereafter, it will appoint a reference individual (typically the household head) to each household, whose age is determined via conditional sampling. Subsequently, the ages of remaining household constituents are inferred using the population age distribution and the household age-mixing contact matrix. Each column of the contact matrix represents the age distribution of contacts in a household setting for the individual within a specific age group. Based on the age of the reference individual, the algorithm performs conditional sampling to assign the ages of other members, thereby constructing a complete household structure.

### 4.4 Schools contact layer

The algorithm incorporates age-specific enrollment rates, school sizes, and student-to-staff ratios to generate school contact networks. It begins by selecting a reference student and determining their school type, then predicts the age distribution of other students using the school age-mixing contact matrix. To simulate the phenomenon of neighboring children attending the same school, students are selected from the household list. Teachers and non-teaching staff (e.g., administrative personnel and janitors) are extracted from the workforce and assigned to schools. This assignment aligns with manually specified student-to-teacher and student-to-staff ratios.

School types are categorized into five types based on official census data: pre-kindergarten, elementary school, middle school, high school, and university. For universities, where a fully connected contact network is unrealistic, close contacts for each individual are modeled as a randomly selected subset of peers within the school. The size of this subset, n, is determined by a Poisson distribution with a default parameter of λ_s_=20.

The mixing pattern within schools is defined by the **sc_mixing_type** attribute, which includes three types: **random** (random mixing), **age_clustered** (age-based mixing), and **age_and_grade_clustered** (mixing based on age and grade). The model defaults to the age-based mixing (**age_clustered**) mode.

### 4.5 Workplaces and community contact layer

The workforce is assigned based on age-specific employment rates, with non-teaching staff distributed to workplaces according to enterprise size. A reference employee is first randomly selected, and their colleagues are allocated using the workforce age-mixing pattern. To reflect the mixing of adults from various living communities in the workplace, workers are chosen at random from the entire population. Similar to universities, the contact network in large workplaces is not fully connected. Close contacts for each employee are modeled as n randomly selected coworkers, where n is generated by a Poisson distribution with λ_w_=20.

At the community level (e.g., parks, shopping malls, community centers, and public transport), the number of close contacts n is also generated using a Poisson distribution, with a parameter λ_c_=20.

### 4.6 Technical validation

We validated our synthetic population against relevant official census statistics (see Supplementary Tables S2-S9 for detailed breakdowns) across multiple dimensions: population, household, school, and workplace. Our validation examined key indicators including age distribution, household size distribution, age-specific school enrollment and employment rates, school size distribution, and workplace size distribution. Specifically, based on 100 synthetic contact networks generated for each country, we computed the mean and the 95 % confidence interval for each of the six aforementioned metrics, with the validation results presented in Figs 2–7.

## Limitations

**Synthetic Nature:** These datasets are synthetic and do not originate from empirical observations.

**Personalized Attributes:** These datasets currently lack customizable individual attributes (e.g., occupation) and detailed scenarios such as long-term care facilities.

**Applicability to Low-Income Countries:** Based on parameters from 13 middle- and high-income countries, these datasets are not recommended for low-income settings, as structural differences may distort disease dynamics. It may serve only as a reference for low-income countries with similar demographic profiles.

**Static Nature:** These datasets are static and lack temporal or spatial detail, making them unsuitable for geographical sensitivity analyses such as localized lockdowns. The framework could be extended to a dynamic network if detailed spatiotemporal data become available. Under the current static configuration, it may somewhat overestimate epidemiological incidence.

**Limited Validation:** Due to the lack of real-world interpersonal contact data for comparison, these datasets were validated using only six macro-demographic indicators, rather than through detailed statistical testing of network topological equivalence.

**Data Quality:** The input parameters used to generate these datasets vary in quality, granularity, and temporal coverage. We recommend users consult the complete parameter set in the “Input Data” folder of our GitHub repository and the parameter timing information in Supplementary Table S10 to assess the dataset’s suitability before use. If higher-quality parameters become available in the future, the synthetic networks can be updated accordingly to improve their accuracy.

## Ethics Statement

The authors have read and followed the ethical requirements for publication in Data in Brief and confirm that the current work does not involve human subjects, animal experiments, or any data collected from social media platforms.

## Credit Author Statement

**Zhilu Yuan:** Conceptualization, Methodology, Formal analysis, Validation, Visualization, Funding acquisition, Writing – original draft. **Ziyao Luo:** Formal analysis, Validation, Visualization, Writing – review & editing, Writing – original draft. **Shenyao Lin:** Formal analysis, Validation, Visualization, Writing – original draft. **Yifang Ma:** Formal analysis, Validation, Visualization, Writing – original draft. **Yushuang Chen:** Data curation, Validation. **Mingda Xu:** Conceptualization, Software, Data curation, Validation, Writing – review & editing. **Zhanwei Du:** Conceptualization, Methodology, Data curation, Validation, Funding acquisition, Writing – review & editing. **Yuan Bai:** Conceptualization, Data curation, Validation, Writing – review & editing.

## Data Availability

FigshareHigh-resolution human contact networks in thirteen countries for infectious disease transmission (Original data).

## Appendix Supplementary materials

Image, application 1
