# Supplementary material for: High-resolution datasets of synthetic human contact network in 13 countries for infectious disease transmission
Source: Data Brief. 2025 Dec 9;64:112373. doi: 10.1016/j.dib.2025.112373 (PMC12813305; doi:10.1016/j.dib.2025.112373)
Supplement: Supplementary file 1 [file mmc1.docx]

**High-resolution datasets of synthetic human contact network in 13 countries for infectious disease transmission**

Zhilu Yuan, Ziyao Luo, Shenyao Lin, Yifang Ma, Yushuang Chen, Mingda Xu, Zhanwei Du & Yuan Bai

This Supplementary material provides additional details of the age-mixing matrices, data sources, and the nine key parameters required for generating synthetic contact networks[[1]](https://paperpile.com/c/Zxqofu/1phQB). The complete raw input data can be accessed directly at: <https://github.com/DEEMOALICE/SynConNet> (Folder “Input Data”).

**Model parameters**

We employ the age-mixing matrices derived by Prem et al. (2017)[[2]](https://paperpile.com/c/Zxqofu/wOFDq) as SynthPops model contact patterns **(Supplementary Table S1)**. Birth rate **(Supplementary Table S2)** is used to simulate population growth within a specific region. Death rate **(Supplementary Table S2)** is used to simulate population reduction in a specific region. Population age distribution **(Supplementary Table S3)** reflects the characteristics of the population's age structure. Household size distribution **(Supplementary Table S4)** is used to simulate household composition. Household age distribution by family size **(Supplementary Table S5)** is used to simulate the age of the household head in different household compositions. School size distribution **(Supplementary Table S6)** is used to model the capacity of educational institutions. Work size distribution **(Supplementary Table S7)** is used to simulate work environments. Employment rate in workplaces **(Supplementary Table S8)** is used to simulate workforce allocation. Enrollment rate in schools **(Supplementary Table S9)** is used to allocate students, teachers, and non - teaching staff. **Supplementary Table S10** records the reference time for all parameters in each country.

**Supplementary Tables**

**Supplementary Table S1. Age-mixing matrices for synthetic contact networks**

| Parameter | Country | Value | Year, Source |
| --- | --- | --- | --- |
| Age-mixing matrices | All countries | See reference for details | 2017[[2]](https://paperpile.com/c/Zxqofu/wOFDq) |

**Supplementary Table S2. Birth rate and Death rate**

| Parameter | Country | Value | Year, Source |
| --- | --- | --- | --- |
| Birth rate | All countries | See reference for details | 2022[[3]](https://paperpile.com/c/Zxqofu/yx2hK) |
| Death rate | All countries | See reference for details | 2022[[4]](https://paperpile.com/c/Zxqofu/UrAiA) |

**Supplementary Table S3. Population age distribution**

| Country  Age Group | Australia[[5]](https://paperpile.com/c/Zxqofu/fy0g) | Canada[[6]](https://paperpile.com/c/Zxqofu/BYJW6) | France[[7]](https://paperpile.com/c/Zxqofu/O1Id) | Germany[[8]](https://paperpile.com/c/Zxqofu/tuB85) | Ireland[[9]](https://paperpile.com/c/Zxqofu/MQQjZ) | Israel[[10]](https://paperpile.com/c/Zxqofu/QXC2A) |
| --- | --- | --- | --- | --- | --- | --- |
| 0-4y | 0.066 | 0.050 | 0.055 | 0.047 | 0.059 | 0.101 |
| 5-9y | 0.064 | 0.056 | 0.059 | 0.047 | 0.065 | 0.095 |
| 10-14y | 0.064 | 0.058 | 0.061 | 0.045 | 0.071 | 0.085 |
| 15-19y | 0.060 | 0.054 | 0.060 | 0.046 | 0.066 | 0.079 |
| 20-24y | 0.061 | 0.060 | 0.057 | 0.053 | 0.064 | 0.072 |
| 25-29y | 0.068 | 0.066 | 0.056 | 0.058 | 0.059 | 0.068 |
| 30-34y | 0.073 | 0.068 | 0.060 | 0.066 | 0.062 | 0.066 |
| 35-39y | 0.072 | 0.068 | 0.062 | 0.064 | 0.071 | 0.064 |
| 40-44y | 0.064 | 0.065 | 0.060 | 0.062 | 0.080 | 0.062 |
| 45-49y | 0.065 | 0.062 | 0.066 | 0.058 | 0.074 | 0.056 |
| 50-54y | 0.061 | 0.064 | 0.067 | 0.073 | 0.066 | 0.047 |
| 55-59y | 0.060 | 0.072 | 0.066 | 0.082 | 0.060 | 0.044 |
| 60-64y | 0.056 | 0.070 | 0.061 | 0.073 | 0.054 | 0.041 |
| 65-69y | 0.049 | 0.060 | 0.058 | 0.060 | 0.046 | 0.039 |
| 70-74y | 0.043 | 0.050 | 0.055 | 0.051 | 0.039 | 0.032 |
| ≥75y | 0.074 | 0.080 | 0.097 | 0.113 | 0.066 | 0.048 |

Note: The notation "#–#y" denotes ages from # to # years, while "≥#y" represents ages # years or older.

**Supplementary Table S3. Population age distribution (continued)**

| Country  Age Group | Italy[[11]](https://paperpile.com/c/Zxqofu/abWC5) | Japan  [[12]](https://paperpile.com/c/Zxqofu/jOqo0) | Spain  [[13]](https://paperpile.com/c/Zxqofu/yVLx2) | Sweden  [[14]](https://paperpile.com/c/Zxqofu/wXmAR) | The  Netherlands  [[15]](https://paperpile.com/c/Zxqofu/3L2HG) | | | U.K.  [[16,17]](https://paperpile.com/c/Zxqofu/oYbiN+fOMh3) | U.S.  [[18]](https://paperpile.com/c/Zxqofu/KJbLk) |
| --- | --- | --- | --- | --- | --- | --- | --- | --- | --- |
| 0-4y | 0.036 | 0.037 | 0.037 | 0.056 | | 0.049 | 0.062 | | 0.061 |
| 5-9y | 0.043 | 0.041 | 0.045 | 0.060 | | 0.051 | 0.056 | | 0.061 |
| 10-14y | 0.048 | 0.043 | 0.052 | 0.060 | | 0.054 | 0.058 | | 0.057 |
| 15-19y | 0.049 | 0.046 | 0.054 | 0.057 | | 0.058 | 0.056 | | 0.056 |
| 20-24y | 0.050 | 0.048 | 0.052 | 0.055 | | 0.064 | 0.063 | | 0.061 |
| 25-29y | 0.051 | 0.049 | 0.053 | 0.066 | | 0.065 | 0.068 | | 0.084 |
| 30-34y | 0.055 | 0.053 | 0.057 | 0.073 | | 0.065 | 0.066 | | 0.085 |
| 35-39y | 0.058 | 0.059 | 0.062 | 0.065 | | 0.061 | 0.067 | | 0.077 |
| 40-44y | 0.066 | 0.067 | 0.076 | 0.061 | | 0.059 | 0.073 | | 0.070 |
| 45-49y | 0.078 | 0.078 | 0.084 | 0.063 | | 0.062 | 0.073 | | 0.070 |
| 50-54y | 0.081 | 0.069 | 0.080 | 0.064 | | 0.073 | 0.064 | | 0.068 |
| 55-59y | 0.080 | 0.063 | 0.075 | 0.063 | | 0.072 | 0.057 | | 0.065 |
| 60-64y | 0.068 | 0.059 | 0.067 | 0.055 | | 0.066 | 0.060 | | 0.059 |
| 65-69y | 0.060 | 0.066 | 0.056 | 0.052 | | 0.058 | 0.048 | | 0.045 |
| 70-74y | 0.057 | 0.073 | 0.048 | 0.051 | | 0.054 | 0.039 | | 0.031 |
| ≥75y | 0.121 | 0.148 | 0.102 | 0.100 | | 0.089 | 0.078 | | 0.050 |

Note: The notation "#–#y" denotes ages from # to # years, while "≥#y" represents ages # years or older.

**Supplementary Table S4. Household size distribution**

| Country  Family size | Australia  [[19]](https://paperpile.com/c/Zxqofu/5guJn) | Canada  [[6]](https://paperpile.com/c/Zxqofu/BYJW6) | France  [[20]](https://paperpile.com/c/Zxqofu/HUNlY) | Germany  [[21]](https://paperpile.com/c/Zxqofu/yFRcV) | | Ireland  [[22]](https://paperpile.com/c/Zxqofu/fX7mP) | Israel[[23]](https://paperpile.com/c/Zxqofu/Tem4n) | |
| --- | --- | --- | --- | --- | --- | --- | --- | --- |
| 1 | 0.256 | 0.294 | 0.382 | | 0.423 | 0.237 | | 0.199 |
| 2 | 0.335 | 0.342 | 0.324 | | 0.332 | 0.290 | | 0.249 |
| 3 | 0.159 | 0.147 | 0.130 | | 0.119 | 0.179 | | 0.141 |
| 4 | 0.154 | 0.134 | 0.109 | | 0.091 | 0.162 | | 0.160 |
| ≥5 | 0.096 | 0.084 | 0.055 | | 0.035 | 0.132 | | 0.251 |

Note: The notation "#" denotes households with # people, while "≥#" represents households with # people or more.

**Supplementary Table S4. Household size distribution (continued)**

| Country  Family size | Italy  [[24]](https://paperpile.com/c/Zxqofu/anpaa) | Japan  [[12]](https://paperpile.com/c/Zxqofu/jOqo0) | Spain  [[25]](https://paperpile.com/c/Zxqofu/NnSos) | Sweden  [[26]](https://paperpile.com/c/Zxqofu/pWvNg) | The  Netherlands  [[27]](https://paperpile.com/c/Zxqofu/Bwbby) | U.K.  [[28]](https://paperpile.com/c/Zxqofu/VsFs1) | U.S.  [[22]](https://paperpile.com/c/Zxqofu/fX7mP) |
| --- | --- | --- | --- | --- | --- | --- | --- |
| 1 | 0.332 | 0.380 | 0.232 | 0.407 | 0.341 | 0.296 | 0.278 |
| 2 | 0.277 | 0.281 | 0.301 | 0.301 | 0.315 | 0.341 | 0.344 |
| 3 | 0.189 | 0.166 | 0.217 | 0.115 | 0.150 | 0.158 | 0.158 |
| 4 | 0.152 | 0.119 | 0.185 | 0.115 | 0.126 | 0.142 | 0.137 |
| ≥5 | 0.051 | 0.055 | 0.065 | 0.062 | 0.068 | 0.063 | 0.083 |

Note: The notation "#" denotes households with # people, while "≥#" represents households with # people or more.

**Supplementary Table S5. Household head age distribution by family size**

| Country | Age  Family  size | 0-29y  (15-34y) | 30-39y  (35-44y) | 40-49y  (45-54y) | 50-59y  (55-64y) | 60-69y  (65-74y) | ≥70y  (≥75y) |
| --- | --- | --- | --- | --- | --- | --- | --- |
| Australia[[25]](https://paperpile.com/c/Zxqofu/NnSos) | 1 | 225482 | 284827 | 259071 | 373084 | 470654 | 961268 |
|  | 2 | 404877 | 404109 | 267442 | 503925 | 698445 | 805230 |
|  | 3 | 168441 | 350739 | 315694 | 340461 | 189945 | 101660 |
|  | 4 | 93956 | 411935 | 523802 | 301020 | 75356 | 26626 |
|  | ≥5 | 62685 | 279695 | 345746 | 159234 | 33124 | 11976 |
| Canada[[29]](https://paperpile.com/c/Zxqofu/zEZWh) | 1 | 692320 | 430545 | 591220 | 802150 | 701620 | 696920 |
|  | 2 | 837115 | 412850 | 673485 | 1158190 | 1024595 | 635915 |
|  | 3 | 419595 | 450410 | 551595 | 432655 | 167490 | 80015 |
|  | 4 | 289260 | 666725 | 621925 | 253400 | 56935 | 21990 |
|  | ≥5 | 151410 | 411540 | 363765 | 143715 | 42115 | 16835 |
| France[[30]](https://paperpile.com/c/Zxqofu/orEUJ) | 1 | 1311466 | 1072312 | 1129593 | 1516165 | 1723951 | 3755587 |
|  | 2 | 859926 | 815962 | 855015 | 1792185 | 2403136 | 2853394 |
|  | 3 | 328540 | 926209 | 1036221 | 988633 | 408270 | 238892 |
|  | 4 | 138444 | 1067274 | 1377243 | 568274 | 121008 | 46410 |
|  | ≥5 | 47925 | 484300 | 776410 | 283062 | 63167 | 21176 |
| Germany[[25]](https://paperpile.com/c/Zxqofu/NnSos) | 1 | 2383621 | 1770736 | 2069720 | 1926422 | 1670689 | 8696744 |
|  | 2 | 993626 | 1134268 | 1629393 | 2412263 | 2756540 | 10281835 |
|  | 3 | 333436 | 971126 | 1558672 | 1336407 | 595381 | 3220595 |
|  | 4 | 128315 | 865007 | 1680719 | 796532 | 182254 | 1895800 |
|  | ≥5 | 49254 | 349937 | 762925 | 353560 | 113745 | 903903 |
| lreland[[22]](https://paperpile.com/c/Zxqofu/fX7mP) | 1 | 35194 | 65253 | 57439 | 63504 | 66894 | 103716 |
|  | 2 | 75992 | 93293 | 49489 | 69185 | 94685 | 96627 |
|  | 3 | 42084 | 77197 | 52639 | 59579 | 41271 | 23240 |
|  | 4 | 20600 | 79235 | 87674 | 55860 | 18523 | 6244 |
|  | ≥5 | 9654 | 55781 | 94181 | 45855 | 10422 | 2898 |
| Israel[[31]](https://paperpile.com/c/Zxqofu/IXIf5) | 1 | 98838 | 68352 | 40019 | 56640 | 72584 | 149727 |
|  | 2 | 96712 | 62517 | 37909 | 93943 | 132954 | 140806 |
|  | 3 | 61316 | 82277 | 53818 | 93387 | 49154 | 20024 |
|  | 4 | 42335 | 135262 | 93831 | 77743 | 20199 | 5883 |
|  | ≥5 | 32742 | 176509 | 209314 | 87373 | 16018 | 5122 |
| ltaly[[25]](https://paperpile.com/c/Zxqofu/NnSos) | 1 | 487750 | 1066168 | 1113297 | 970313 | 1057882 | 2972263 |
|  | 2 | 219456 | 695564 | 716950 | 856232 | 1530358 | 2648396 |
|  | 3 | 145823 | 837342 | 1110279 | 1132583 | 953711 | 712578 |
|  | 4 | 54893 | 689634 | 1506364 | 1086133 | 427901 | 212476 |
|  | ≥5 | 19411 | 197795 | 502579 | 377794 | 169207 | 142158 |
| Japan[[12]](https://paperpile.com/c/Zxqofu/jOqo0) | 1 | 3765207 | 2294023 | 2308879 | 2517705 | 2476914 | 5385711 |
|  | 2 | 596151 | 1056748 | 1510415 | 2335949 | 3370891 | 6709724 |
|  | 3 | 290393 | 1140687 | 1710913 | 1939921 | 1809845 | 2300090 |
|  | 4 | 131584 | 1264434 | 2282369 | 1487721 | 734866 | 697799 |
|  | ≥5 | 39606 | 541015 | 1000268 | 552735 | 358022 | 531641 |
| Spain[[32]](https://paperpile.com/c/Zxqofu/tVZKw) | 1 | 64293 | 113416 | 95043 | 116760 | 146680 | 330635 |
|  | 2 | 84686 | 147509 | 112912 | 210194 | 164053 | 399604 |
|  | 3 | 56415 | 235902 | 226690 | 222079 | 135818 | 88877 |
|  | 4 | 19679 | 164919 | 258270 | 146621 | 50090 | 31487 |
|  | ≥5 | 7313 | 45037 | 86425 | 62616 | 32350 | 28753 |
| Sweden[[33]](https://paperpile.com/c/Zxqofu/Qxi2j) | 1 | 179614 | 132213 | 113400 | 120705 | 122494 | 211914 |
|  | 2 | 53898 | 65208 | 69011 | 108289 | 172998 | 155798 |
|  | 3 | 23398 | 63749 | 68277 | 75921 | 35259 | 14005 |
|  | 4 | 8877 | 81336 | 116649 | 60751 | 12023 | 3680 |
|  | ≥5 | 1622 | 36349 | 74601 | 30715 | 7174 | 4223 |
| The Netherlands  [[32]](https://paperpile.com/c/Zxqofu/tVZKw) | 1 | 208819 | 229761 | 224804 | 252908 | 231841 | 463899 |
|  | 2 | 129610 | 145967 | 160660 | 290399 | 357944 | 408763 |
|  | 3 | 60722 | 162139 | 187875 | 175829 | 73614 | 48001 |
|  | 4 | 25171 | 182263 | 241850 | 115664 | 21581 | 10490 |
|  | ≥5 | 9125 | 84476 | 143003 | 63860 | 13503 | 10061 |
| U.K.[[34]](https://paperpile.com/c/Zxqofu/mfOz2) | 1 | 474100 | 723400 | 814400 | 1391300 | 1536000 | 3258000 |
|  | 2 | 1090100 | 1122100 | 925700 | 1780800 | 2120200 | 2569600 |
|  | 3 | 466800 | 1056800 | 1025300 | 1065900 | 450900 | 221000 |
|  | 4 | 293000 | 1124400 | 1403500 | 848400 | 167000 | 44900 |
|  | ≥5 | 908014 | 510300 | 712400 | 382500 | 97600 | 23099 |
| U.S.[[22]](https://paperpile.com/c/Zxqofu/fX7mP) | 1 | 13516498 | 4692256 | 4342088 | 4482155 | 4096970 | 38866869 |
|  | 2 | 16093922 | 5587009 | 5170068 | 5336845 | 4878209 | 4628045 |
|  | 3 | 7620529 | 2645468 | 2448045 | 2527015 | 2309849 | 2191396 |
|  | 4 | 6387566 | 2217445 | 2051964 | 2118157 | 1936128 | 1836839 |
|  | ≥5 | 5333071 | 1851377 | 1713214 | 1768480 | 1616500 | 1533603 |

Note: The notation "#–#y" denotes ages from # to # years, while "≥#y" represents ages # years or older. The household head age groups for Canada are "15-34y, 35-44y, 45-54y, 55-64y, 65-74y, ≥75y" in brackets, while those for the rest of the countries are "0-29y, 30-39y, 40-49y, 50-59y, 60-69y, ≥70y". The notation "#" denotes households with # people, while "≥#" represents households with # people or more. Due to the lack of data for Sweden, we imputed the values from Norway.

**Supplementary Table S6. School size distribution**

| Number of people  in school  Country | 1-100 | 101-999 | ≥1000 |
| --- | --- | --- | --- |
| Australia[[35]](https://paperpile.com/c/Zxqofu/DLUMp) | 0.623 | 0.305 | 0.072 |
| Canada[[36–38]](https://paperpile.com/c/Zxqofu/m1DVP+VykEx+5JTN7) | 0.663 | 0.138 | 0.199 |
| France[[39]](https://paperpile.com/c/Zxqofu/nsr8h) | 0.025 | 0.963 | 0.012 |
| Germany[[25]](https://paperpile.com/c/Zxqofu/NnSos) | 0.025 | 0.963 | 0.012 |
| Ireland[[40]](https://paperpile.com/c/Zxqofu/K2CN1) | 0.000 | 0.994 | 0.006 |
| Israel[[41–43]](https://paperpile.com/c/Zxqofu/ZnzyV+0bos3+lVk1S) | 0.028 | 0.967 | 0.005 |
| Italy[[44,45]](https://paperpile.com/c/Zxqofu/HeoTJ+NNFmA) | 0.418 | 0.581 | 0.001 |
| Japan[[46]](https://paperpile.com/c/Zxqofu/xW3kD) | 0.025 | 0.962 | 0.013 |
| Spain[[47]](https://paperpile.com/c/Zxqofu/7H6xu) | 0.016 | 0.981 | 0.003 |
| Sweden[[48–50]](https://paperpile.com/c/Zxqofu/5E6Ma+Yf5wF+GfWw5) | 0.630 | 0.365 | 0.005 |
| The Netherlands[[51]](https://paperpile.com/c/Zxqofu/hebJS) | 0.160 | 0.772 | 0.068 |
| U.K.[[39]](https://paperpile.com/c/Zxqofu/nsr8h) | 0.025 | 0.963 | 0.012 |
| U.S.[[52]](https://paperpile.com/c/Zxqofu/mUeP7) | 0.953 | | 0.047 |

Note: The symbol “#-#” indicates that the school's capacity ranges from # to # individuals, while “≥#” means the school's capacity exceeds # individuals. Due to the absence of detailed U.S. data on school sizes between 1 and 100 students, the school size bins are defined as 1-999 and ≥1000. We assigned the values from the UK to Germany and France, and those from Norway to Sweden, due to the lack of data for the former countries.

**Supplementary Table S7. Work size distribution**

| Number of people  in workplace  Country | 1-9 | 10-49 | 50-249 | ≥250 |
| --- | --- | --- | --- | --- |
| Australia[[53]](https://paperpile.com/c/Zxqofu/yoeNc) | 0.596 | 0.379 | 0.023 | 0.002 |
| Canada[[54]](https://paperpile.com/c/Zxqofu/GYul8) | 0.111 | 0.196 | 0.154 | 0.539 |
| France[[55]](https://paperpile.com/c/Zxqofu/fqZ5v) | 0.956 | 0.037 | 0.006 | 0.001 |
| Germany[[25]](https://paperpile.com/c/Zxqofu/NnSos) | 0.830 | 0.140 | 0.030 | 0.010 |
| Ireland[[56,57]](https://paperpile.com/c/Zxqofu/h7jn1+uOwZD) | 0.697 | 0.057 | 0.010 | 0.236 |
| Israel[[58]](https://paperpile.com/c/Zxqofu/eIOpc) | 0.922 | 0.062 | 0.014 | 0.002 |
| Italy[[24]](https://paperpile.com/c/Zxqofu/anpaa) | 0.860 | 0.121 | 0.016 | 0.003 |
| Japan[[59]](https://paperpile.com/c/Zxqofu/uE1JO) | 0.799 | 0.174 | 0.024 | 0.003 |
| Spain[[60]](https://paperpile.com/c/Zxqofu/vDBMt) | 0.123 | 0.243 | 0.242 | 0.393 |
| Sweden[[61]](https://paperpile.com/c/Zxqofu/AsAYk) | 0.966 | 0.028 | 0.005 | 0.001 |
| The Netherlands[[62]](https://paperpile.com/c/Zxqofu/uI11U) | 0.959 | 0.033 | 0.007 | 0.001 |
| U.K.[[63]](https://paperpile.com/c/Zxqofu/4H3dB) | 0.898 | 0.083 | 0.015 | 0.004 |
| U.S.[[64]](https://paperpile.com/c/Zxqofu/DbPL1) | 0.733 | 0.214 | 0.046 | 0.007 |

Note: The symbol “#-#” indicates that the workplace's capacity ranges from # to # individuals, while “≥#” means the workplace's capacity exceeds # individuals. “/” represents a missing value, indicating the absence of relevant data. These missing values were replaced with zeros.

**Supplementary Table S8. Employment rate in workplaces**

| Country  Age Group | Australia  [[65]](https://paperpile.com/c/Zxqofu/C9O70) | Canada  [[66,67]](https://paperpile.com/c/Zxqofu/b7ZmI+RZWgJ) | France[[65]](https://paperpile.com/c/Zxqofu/C9O70) | Germany  [[68]](https://paperpile.com/c/Zxqofu/olBhA) | Ireland[[65]](https://paperpile.com/c/Zxqofu/C9O70) | Israel[[69]](https://paperpile.com/c/Zxqofu/2vmpv) |
| --- | --- | --- | --- | --- | --- | --- |
| 15y | 0.467 | 0.481 | 0.128 | 0.437 | 0.238 | 0.416 |
| 16y | 0.467 | 0.481 | 0.128 | 0.437 | 0.238 | 0.416 |
| 17y | 0.467 | 0.481 | 0.128 | 0.437 | 0.238 | 0.416 |
| 18y | 0.467 | 0.481 | 0.128 | 0.437 | 0.238 | 0.416 |
| 19y | 0.467 | 0.481 | 0.128 | 0.437 | 0.238 | 0.416 |
| 20y | 0.739 | 0.804 | 0.534 | 0.437 | 0.630 | 0.416 |
| 21y | 0.739 | 0.804 | 0.534 | 0.437 | 0.630 | 0.416 |
| 22y | 0.739 | 0.804 | 0.534 | 0.437 | 0.630 | 0.416 |
| 23y | 0.739 | 0.804 | 0.534 | 0.437 | 0.630 | 0.416 |
| 24y | 0.739 | 0.804 | 0.534 | 0.437 | 0.630 | 0.416 |
| 25y | 0.801 | 0.882 | 0.774 | 0.437 | 0.786 | 0.808 |
| 26y | 0.801 | 0.882 | 0.774 | 0.437 | 0.786 | 0.808 |
| 27y | 0.801 | 0.882 | 0.774 | 0.437 | 0.786 | 0.808 |
| 28y | 0.801 | 0.882 | 0.774 | 0.437 | 0.786 | 0.808 |
| 29y | 0.801 | 0.882 | 0.774 | 0.437 | 0.786 | 0.808 |
| 30y | 0.817 | 0.916 | 0.808 | 0.730 | 0.820 | 0.808 |
| 31y | 0.817 | 0.916 | 0.808 | 0.730 | 0.820 | 0.808 |
| 32y | 0.817 | 0.916 | 0.808 | 0.730 | 0.820 | 0.808 |
| 33y | 0.817 | 0.916 | 0.808 | 0.730 | 0.820 | 0.808 |
| 34y | 0.817 | 0.916 | 0.808 | 0.730 | 0.820 | 0.808 |
| 35y | 0.833 | 0.897 | 0.820 | 0.730 | 0.811 | 0.808 |
| 36y | 0.833 | 0.897 | 0.820 | 0.730 | 0.811 | 0.808 |
| 37y | 0.833 | 0.897 | 0.820 | 0.730 | 0.811 | 0.808 |
| 38y | 0.833 | 0.897 | 0.820 | 0.730 | 0.811 | 0.808 |
| 39y | 0.833 | 0.897 | 0.820 | 0.730 | 0.811 | 0.808 |
| 40y | 0.832 | 0.903 | 0.844 | 0.730 | 0.818 | 0.808 |
| 41y | 0.832 | 0.903 | 0.844 | 0.730 | 0.818 | 0.808 |
| 42y | 0.832 | 0.903 | 0.844 | 0.730 | 0.818 | 0.808 |
| 43y | 0.832 | 0.903 | 0.844 | 0.730 | 0.818 | 0.808 |
| 44y | 0.832 | 0.903 | 0.844 | 0.730 | 0.818 | 0.808 |
| 45y | 0.829 | 0.853 | 0.840 | 0.730 | 0.809 | 0.808 |
| 46y | 0.829 | 0.853 | 0.840 | 0.730 | 0.809 | 0.808 |
| 47y | 0.829 | 0.853 | 0.840 | 0.730 | 0.809 | 0.808 |
| 48y | 0.829 | 0.853 | 0.840 | 0.730 | 0.809 | 0.808 |
| 49y | 0.829 | 0.853 | 0.840 | 0.730 | 0.809 | 0.808 |
| 50y | 0.801 | 0.855 | 0.833 | 0.730 | 0.769 | 0.808 |
| 51y | 0.801 | 0.855 | 0.833 | 0.730 | 0.769 | 0.808 |
| 52y | 0.801 | 0.855 | 0.833 | 0.730 | 0.769 | 0.808 |
| 53y | 0.801 | 0.855 | 0.833 | 0.730 | 0.769 | 0.808 |
| 54y | 0.801 | 0.855 | 0.833 | 0.730 | 0.769 | 0.808 |
| 55y | 0.734 | 0.715 | 0.751 | 0.730 | 0.724 | 0.699 |
| 56y | 0.734 | 0.715 | 0.751 | 0.730 | 0.724 | 0.699 |
| 57y | 0.734 | 0.715 | 0.751 | 0.730 | 0.724 | 0.699 |
| 58y | 0.734 | 0.715 | 0.751 | 0.730 | 0.724 | 0.699 |
| 59y | 0.734 | 0.715 | 0.751 | 0.730 | 0.724 | 0.699 |
| 60y | 0.569 | 0.541 | 0.355 | 0.730 | 0.547 | 0.699 |
| 61y | 0.569 | 0.541 | 0.355 | 0.730 | 0.547 | 0.699 |
| 62y | 0.569 | 0.541 | 0.355 | 0.730 | 0.547 | 0.699 |
| 63y | 0.569 | 0.541 | 0.355 | 0.730 | 0.547 | 0.699 |
| 64y | 0.569 | 0.541 | 0.355 | 0.730 | 0.547 | 0.699 |
| 65y | / | 0.280 | 0.086 | / | 0.263 | / |
| 66y | / | 0.280 | 0.086 | / | 0.263 | / |
| 67y | / | 0.280 | 0.086 | / | 0.263 | / |
| 68y | / | 0.280 | 0.086 | / | 0.263 | / |
| 69y | / | 0.280 | 0.086 | / | 0.263 | / |
| 70y | / | 0.071 | 0.026 | / | 0.136 | / |
| 71y | / | 0.071 | 0.026 | / | 0.136 | / |
| 72y | / | 0.071 | 0.026 | / | 0.136 | / |
| 73y | / | 0.071 | 0.026 | / | 0.136 | / |
| 74y | / | 0.071 | 0.026 | / | 0.136 | / |
| ≥75y | / | 0.071 | 0.012 | / | / | / |

Note: The notation "#y" denotes age # years, while "≥#y" represents ages # years or older. “/” represents a missing value, indicating the absence of relevant data. These missing values were replaced with zeros.

**Supplementary Table S8. Employment rate in workplaces (continued)**

| Country    Age Group | Italy  [[23]](https://paperpile.com/c/Zxqofu/Tem4n) | Japan  [[70]](https://paperpile.com/c/Zxqofu/FwEUS) | Spain  [[60]](https://paperpile.com/c/Zxqofu/vDBMt) | Sweden  [[61]](https://paperpile.com/c/Zxqofu/AsAYk) | The  Netherlands  [[69]](https://paperpile.com/c/Zxqofu/2vmpv) | U.K.  [[65,69]](https://paperpile.com/c/Zxqofu/2vmpv+C9O70) | U.S.  [[69]](https://paperpile.com/c/Zxqofu/2vmpv) |
| --- | --- | --- | --- | --- | --- | --- | --- |
| 15y | 0.065 | 0.467 | 0.230 | 0.215 | 0.649 | / | 0.511 |
| 16y | 0.065 | 0.467 | 0.230 | 0.215 | 0.649 | 0.240 | 0.300 |
| 17y | 0.065 | 0.467 | 0.230 | 0.215 | 0.649 | 0.240 | 0.300 |
| 18y | 0.065 | 0.467 | 0.230 | 0.215 | 0.649 | 0.620 | 0.300 |
| 19y | 0.065 | 0.467 | 0.230 | 0.215 | 0.649 | 0.620 | 0.300 |
| 20y | 0.445 | 0.467 | 0.230 | 0.601 | 0.779 | 0.620 | 0.693 |
| 21y | 0.445 | 0.467 | 0.230 | 0.601 | 0.779 | 0.620 | 0.693 |
| 22y | 0.445 | 0.467 | 0.230 | 0.601 | 0.779 | 0.620 | 0.693 |
| 23y | 0.445 | 0.467 | 0.230 | 0.601 | 0.779 | 0.620 | 0.693 |
| 24y | 0.445 | 0.467 | 0.230 | 0.601 | 0.779 | 0.620 | 0.693 |
| 25y | 0.704 | 0.865 | 0.772 | 0.804 | 0.870 | 0.850 | 0.861 |
| 26y | 0.704 | 0.865 | 0.772 | 0.804 | 0.870 | 0.850 | 0.861 |
| 27y | 0.704 | 0.865 | 0.772 | 0.804 | 0.870 | 0.850 | 0.861 |
| 28y | 0.704 | 0.865 | 0.772 | 0.804 | 0.870 | 0.850 | 0.861 |
| 29y | 0.704 | 0.865 | 0.772 | 0.804 | 0.870 | 0.850 | 0.861 |
| 30y | 0.794 | 0.865 | 0.772 | 0.804 | 0.871 | 0.850 | 0.838 |
| 31y | 0.794 | 0.865 | 0.772 | 0.804 | 0.871 | 0.850 | 0.838 |
| 32y | 0.794 | 0.865 | 0.772 | 0.804 | 0.871 | 0.850 | 0.838 |
| 33y | 0.794 | 0.865 | 0.772 | 0.804 | 0.871 | 0.850 | 0.838 |
| 34y | 0.794 | 0.865 | 0.772 | 0.804 | 0.871 | 0.850 | 0.838 |
| 35y | 0.809 | 0.865 | 0.772 | 0.862 | 0.860 | 0.850 | 0.838 |
| 36y | 0.809 | 0.865 | 0.772 | 0.862 | 0.860 | 0.850 | 0.838 |
| 37y | 0.809 | 0.865 | 0.772 | 0.862 | 0.860 | 0.850 | 0.838 |
| 38y | 0.809 | 0.865 | 0.772 | 0.862 | 0.860 | 0.850 | 0.838 |
| 39y | 0.809 | 0.865 | 0.772 | 0.862 | 0.860 | 0.850 | 0.838 |
| 40y | 0.803 | 0.865 | 0.772 | 0.862 | 0.853 | 0.850 | 0.838 |
| 41y | 0.803 | 0.865 | 0.772 | 0.862 | 0.853 | 0.850 | 0.838 |
| 42y | 0.803 | 0.865 | 0.772 | 0.862 | 0.853 | 0.850 | 0.838 |
| 43y | 0.803 | 0.865 | 0.772 | 0.862 | 0.853 | 0.850 | 0.838 |
| 44y | 0.803 | 0.865 | 0.772 | 0.862 | 0.853 | 0.850 | 0.838 |
| 45y | 0.788 | 0.865 | 0.772 | 0.880 | 0.871 | 0.850 | 0.814 |
| 46y | 0.788 | 0.865 | 0.772 | 0.880 | 0.871 | 0.850 | 0.814 |
| 47y | 0.788 | 0.865 | 0.772 | 0.880 | 0.871 | 0.850 | 0.814 |
| 48y | 0.788 | 0.865 | 0.772 | 0.880 | 0.871 | 0.850 | 0.814 |
| 49y | 0.788 | 0.865 | 0.772 | 0.880 | 0.871 | 0.850 | 0.814 |
| 50y | 0.749 | 0.865 | 0.772 | 0.880 | 0.834 | 0.710 | 0.814 |
| 51y | 0.749 | 0.865 | 0.772 | 0.880 | 0.834 | 0.710 | 0.814 |
| 52y | 0.749 | 0.865 | 0.772 | 0.880 | 0.834 | 0.710 | 0.814 |
| 53y | 0.749 | 0.865 | 0.772 | 0.880 | 0.834 | 0.710 | 0.814 |
| 54y | 0.749 | 0.865 | 0.772 | 0.880 | 0.834 | 0.710 | 0.814 |
| 55y | 0.597 | 0.781 | 0.577 | 0.770 | 0.794 | 0.710 | 0.761 |
| 56y | 0.597 | 0.781 | 0.577 | 0.770 | 0.794 | 0.710 | 0.761 |
| 57y | 0.597 | 0.781 | 0.577 | 0.770 | 0.794 | 0.710 | 0.761 |
| 58y | 0.597 | 0.781 | 0.577 | 0.770 | 0.794 | 0.710 | 0.761 |
| 59y | 0.597 | 0.781 | 0.577 | 0.770 | 0.794 | 0.710 | 0.761 |
| 60y | 0.246 | 0.781 | 0.577 | 0.770 | 0.626 | 0.710 | 0.636 |
| 61y | 0.246 | 0.781 | 0.577 | 0.770 | 0.626 | 0.710 | 0.636 |
| 62y | 0.246 | 0.781 | 0.577 | 0.770 | 0.626 | 0.710 | 0.636 |
| 63y | 0.246 | 0.781 | 0.577 | 0.770 | 0.626 | 0.710 | 0.636 |
| 64y | 0.246 | 0.781 | 0.577 | 0.770 | 0.626 | 0.710 | 0.636 |
| 65y | 0.095 | 0.781 | / | 0.192 | 0.207 | 0.110 | 0.294 |
| 66y | 0.095 | 0.781 | / | 0.192 | 0.207 | 0.110 | 0.294 |
| 67y | 0.095 | 0.781 | / | 0.192 | 0.207 | 0.110 | 0.294 |
| 68y | 0.095 | 0.781 | / | 0.192 | 0.207 | 0.110 | 0.294 |
| 69y | 0.095 | 0.781 | / | 0.192 | 0.207 | 0.110 | 0.294 |
| 70y | 0.048 | 0.781 | / | 0.192 | 0.077 | 0.110 | 0.294 |
| 71y | 0.048 | 0.781 | / | 0.192 | 0.077 | 0.110 | 0.294 |
| 72y | 0.048 | 0.781 | / | 0.192 | 0.077 | 0.110 | 0.294 |
| 73y | 0.048 | 0.781 | / | 0.192 | 0.077 | 0.110 | 0.294 |
| 74y | 0.048 | 0.781 | / | 0.192 | 0.077 | 0.110 | 0.294 |
| ≥75y | 0.003 | 0.781 | / | / | 0.020 | 0.110 | 0.061 |

Note: The notation "#y" denotes age # years, while "≥#y" represents ages # years or older. “/” represents a missing value, indicating the absence of relevant data. These missing values were replaced with zeros.

**Supplementary Table S9. Enrollment rate in schools**

| Country  Age Group | Australia  [[65]](https://paperpile.com/c/Zxqofu/C9O70) | Canada  [[71]](https://paperpile.com/c/Zxqofu/NS0jN) | France[[72]](https://paperpile.com/c/Zxqofu/ejbqW) | Germany  [[65]](https://paperpile.com/c/Zxqofu/C9O70) | Ireland[[65]](https://paperpile.com/c/Zxqofu/C9O70) | Israel[[72]](https://paperpile.com/c/Zxqofu/ejbqW) |
| --- | --- | --- | --- | --- | --- | --- |
| 0-1y | 0.00 | 0.00 | 0.00 | 0.00 | 0.25 | 0.57 |
| 2y | 0.62 | 0.00 | 0.00 | 0.67 | 0.25 | 0.75 |
| 3y | 0.65 | 0.00 | 1.00 | 0.94 | 1.00 | 1.00 |
| 4y | 0.82 | 0.00 | 1.00 | 0.94 | 1.00 | 1.00 |
| 5y | 0.97 | 0.00 | 1.00 | 0.94 | 1.00 | 1.00 |
| 6y | 1.00 | 1.00 | 1.00 | 0.99 | 1.00 | 0.96 |
| 7y | 0.99 | 1.00 | 1.00 | 0.99 | 1.00 | 0.96 |
| 8y | 1.00 | 1.00 | 1.00 | 0.99 | 1.00 | 0.96 |
| 9y | 1.00 | 1.00 | 1.00 | 0.99 | 1.00 | 0.96 |
| 10y | 1.00 | 1.00 | 1.00 | 0.99 | 1.00 | 0.96 |
| 11y | 1.00 | 1.00 | 1.00 | 0.99 | 1.00 | 0.96 |
| 12y | 1.00 | 1.00 | 1.00 | 0.99 | 1.00 | 0.96 |
| 13y | 1.00 | 1.00 | 1.00 | 0.99 | 1.00 | 0.96 |
| 14y | 1.00 | 1.00 | 1.00 | 0.99 | 1.00 | 0.96 |
| 15y | 1.00 | 0.97 | 0.87 | 0.87 | 0.94 | 0.67 |
| 16y | 0.99 | 0.96 | 0.87 | 0.87 | 0.94 | 0.67 |
| 17y | 0.93 | 0.92 | 0.87 | 0.87 | 0.94 | 0.67 |
| 18y | 0.71 | 0.70 | 0.87 | 0.87 | 0.94 | 0.67 |
| 19y | 0.68 | 0.65 | 0.87 | 0.87 | 0.94 | 0.67 |
| 20y | 0.68 | 0.58 | 0.38 | 0.51 | 0.43 | 0.21 |
| 21y | 0.63 | 0.54 | 0.38 | 0.51 | 0.43 | 0.21 |
| 22y | 0.54 | 0.43 | 0.38 | 0.51 | 0.43 | 0.21 |
| 23y | 0.46 | 0.35 | 0.38 | 0.51 | 0.43 | 0.21 |
| 24y | 0.39 | 0.29 | 0.38 | 0.51 | 0.43 | 0.21 |
| 25y | 0.32 | 0.17 | 0.08 | 0.20 | 0.12 | 0.19 |
| 26y | 0.28 | 0.15 | 0.08 | 0.20 | 0.12 | 0.19 |
| 27y | 0.25 | 0.11 | 0.08 | 0.20 | 0.12 | 0.19 |
| 28y | 0.22 | 0.10 | 0.08 | 0.20 | 0.12 | 0.19 |
| 29y | 0.21 | 0.09 | 0.08 | 0.20 | 0.12 | 0.19 |
| 30-34y | 0.16 | 0.06 | 0.02 | 0.08 | 0.07 | 0.07 |
| 35-39y | 0.13 | 0.04 | 0.01 | 0.03 | 0.05 | 0.04 |
| 40-50y | 0.06 | 0.01 | 0.00 | 0.01 | 0.03 | 0.01 |
| 51-64y | 0.06 | 0.01 | 0.00 | 0.01 | 0.03 | 0.01 |
| ≥65y | / | / | 0.00 | / | / | / |

Note: The notation "#y" denotes age # years, "#–#y" denotes ages from # to # years, while "≥#y" represents ages # years or older. “/” represents a missing value, indicating the absence of relevant data. These missing values were replaced with zeros.

**Supplementary Table S9. Enrollment rate in schools (continued)**

| Country  Age Group | | Italy[[65]](https://paperpile.com/c/Zxqofu/C9O70) | | Japan[[73]](https://paperpile.com/c/Zxqofu/PbmNq) | | Spain[[65]](https://paperpile.com/c/Zxqofu/C9O70) | | Sweden  [[65]](https://paperpile.com/c/Zxqofu/C9O70) | | The  Netherlands  [[65]](https://paperpile.com/c/Zxqofu/C9O70) | U.K.  [[72]](https://paperpile.com/c/Zxqofu/ejbqW) | | U.S.[[65]](https://paperpile.com/c/Zxqofu/C9O70) |
| --- | --- | --- | --- | --- | --- | --- | --- | --- | --- | --- | --- | --- | --- |
| 0-1y | 0.05 | | 0.03 | | 0.41 | | 0.473 | | 0 | | 0 | 0 | |
| 2y | 0.15 | | 0.08 | | 0.63 | | 0.473 | | 0 | | 0 | 0 | |
| 3y | 0.95 | | 0.95 | | 0.97 | | 0.945 | | 0.89 | | 1.00 | 0.529 | |
| 4y | 0.95 | | 0.95 | | 0.97 | | 0.945 | | 0.89 | | 1.00 | 0.529 | |
| 5y | 0.95 | | 0.95 | | 0.97 | | 0.945 | | 0.89 | | 0.96 | 0.95 | |
| 6y | 0.99 | | 1.00 | | 0.98 | | 0.992 | | 1.00 | | 0.97 | 0.95 | |
| 7y | 0.99 | | 1.00 | | 0.98 | | 0.992 | | 1.00 | | 0.97 | 0.95 | |
| 8y | 0.99 | | 1.00 | | 0.98 | | 0.992 | | 1.00 | | 0.97 | 0.95 | |
| 9y | 0.99 | | 1.00 | | 0.98 | | 0.992 | | 1.00 | | 0.97 | 0.95 | |
| 10y | 0.99 | | 1.00 | | 0.98 | | 0.992 | | 1.00 | | 0.97 | 0.987 | |
| 11y | 0.99 | | 1.00 | | 0.98 | | 0.992 | | 1.00 | | 0.97 | 0.987 | |
| 12y | 0.99 | | 1.00 | | 0.98 | | 0.992 | | 1.00 | | 0.97 | 0.987 | |
| 13y | 0.99 | | 1.00 | | 0.98 | | 0.992 | | 1.00 | | 0.97 | 0.987 | |
| 14y | 0.99 | | 1.00 | | 0.98 | | 0.992 | | 1.00 | | 0.97 | 0.987 | |
| 15y | 0.86 | | 1.00 | | 0.87 | | 0.880 | | 0.93 | | 0.99 | 0.977 | |
| 16y | 0.86 | | 0.99 | | 0.87 | | 0.880 | | 0.93 | | 0.97 | 0.977 | |
| 17y | 0.86 | | 0.97 | | 0.87 | | 0.880 | | 0.93 | | 0.92 | 0.977 | |
| 18y | 0.86 | | 0.806 | | 0.87 | | 0.880 | | 0.93 | | 0.69 | 0.793 | |
| 19y | 0.86 | | 0.806 | | 0.87 | | 0.880 | | 0.93 | | 0.62 | 0.793 | |
| 20y | 0.37 | | 0.806 | | 0.46 | | 0.4372 | | 0.53 | | 0.57 | 0.409 | |
| 21y | 0.37 | | 0.806 | | 0.46 | | 0.4372 | | 0.53 | | 0.43 | 0.409 | |
| 22y | 0.37 | | 0.806 | | 0.46 | | 0.4372 | | 0.53 | | 0.29 | 0.409 | |
| 23y | 0.37 | | 0.806 | | 0.46 | | 0.4372 | | 0.53 | | 0.2 | 0.409 | |
| 24y | 0.37 | | 0.806 | | 0.46 | | 0.4372 | | 0.53 | | 0.16 | 0.409 | |
| 25y | 0.13 | | 0.65 | | 0.16 | | 0.258 | | 0.17 | | 0.13 | 0.113 | |
| 26y | 0.13 | | 0.65 | | 0.16 | | 0.258 | | 0.17 | | 0.11 | 0.113 | |
| 27y | 0.13 | | 0.65 | | 0.16 | | 0.258 | | 0.17 | | 0.09 | 0.113 | |
| 28y | 0.13 | | 0.65 | | 0.16 | | 0.258 | | 0.17 | | 0.08 | 0.113 | |
| 29y | 0.13 | | 0.65 | | 0.16 | | 0.258 | | 0.17 | | 0.08 | 0.113 | |
| 30-34y | 0.05 | | 0.65 | | 0.07 | | 0.177 | | 0.07 | | 0.06 | 0.113 | |
| 35-39y | 0.03 | | / | | 0.05 | | 0.133 | | 0.04 | | 0.05 | 0.027 | |
| 40-50y | 0.01 | | / | | 0.01 | | 0.049 | | 0.02 | | 0.02 | 0.027 | |
| 51-64y | 0.01 | | / | | 0.01 | | 0.049 | | 0.02 | | 0.02 | 0 | |
| ≥65y | / | | / | | / | | / | | / | | 0.02 | 0 | |

Note: The notation "#y" denotes age # years, "#–#y" denotes ages from # to # years, while "≥#y" represents ages # years or older. “/” represents a missing value, indicating the absence of relevant data. These missing values were replaced with zeros.

**Supplementary Table S10. Reference years for the collected parameters by country**

| Country | 1 | 2 | 3 | 4 | 5 | 6 | 7 | 8 | 9 | earliest | latest | span |
| --- | --- | --- | --- | --- | --- | --- | --- | --- | --- | --- | --- | --- |
| Australia | 2021 | 2021 | 2021 | 2023 | 2023 | 2020 | 2020 | 2022 | 2022 | 2020 | 2023 | 3 |
| Canada | 2021 | 2021 | 2017 | 2022 | 2023 | 2023 | 2022 | 2022 | 2022 | 2017 | 2023 | 6 |
| France | 2021 | 2021 | 2015 | 2019 | 2018 | 2021 | 2019 | 2022 | 2022 | 2015 | 2022 | 7 |
| Germany | 2022 | 2019 | 2014 | 2019 | 2020 | 2022 | 2019 | 2022 | 2022 | 2014 | 2022 | 8 |
| Ireland | 2022 | 2012 | 2012 | 2020 | 2022 | 2021 | 2019 | 2022 | 2022 | 2012 | 2022 | 10 |
| Israel | 2020 | 2020 | 2022 | 2020 | 2016 | 2022 | 2020 | 2022 | 2022 | 2016 | 2022 | 6 |
| Italy | 2022 | 2022 | 2012 | 2021 | 2021 | 2014 | 2020 | 2022 | 2022 | 2012 | 2022 | 10 |
| Japan | 2020 | 2020 | 2020 | 2022 | 2019 | 2022 | 2015 | 2022 | 2022 | 2015 | 2022 | 7 |
| Spain | 2023 | 2013 | 2013 | 2022 | 2021 | 2022 | 2020 | 2022 | 2022 | 2013 | 2023 | 10 |
| Sweden | 2022 | 2022 | 2014 | 2022 | 2020 | 2021 | 2019 | 2022 | 2022 | 2014 | 2022 | 8 |
| The Netherlands | 2022 | 2018 | 2015 | 2021 | 2021 | 2021 | 2021 | 2022 | 2022 | 2015 | 2022 | 7 |
| U.K. | 2018 | 2021 | 2019 | 2019 | 2015 | 2022 | 2019 | 2022 | 2022 | 2015 | 2022 | 7 |
| U.S. | 2017 | 2019 | 2019 | 2020 | 2019 | 2019 | 2020 | 2022 | 2022 | 2017 | 2022 | 5 |

Note: “1” represents population age distribution, “2” represents household size distribution, “3” represents household head age distribution by family size, “4” represents school size distribution, “5” represents workplace size distribution, “6” represents employment rate in workplaces, “7” represents enrollment rate in schools, “8” represents birth rate, “9” represents death rate.

**References**

[1] Z. Yuan, Z. Luo, S. Lin, Y. Ma, Y. Chen, M. Xu, Y. Li, High-resolution datasets of synthetic human contact network in 13 countries for infectious disease transmission [dataset], figshare, 2025. https://doi.org/10.6084/m9.figshare.28466948[.](http://dx.doi.org/10.6084/m9.figshare.28466948.)

[2] [K. Prem, A.R. Cook, M. Jit, Projecting social contact matrices in 152 countries using contact surveys and demographic data, PLoS Comput Biol 13 (2017) e1005697. https://doi.org/](http://paperpile.com/b/Zxqofu/wOFDq)[10.1371/journal.pcbi.1005697.](http://dx.doi.org/10.1371/journal.pcbi.1005697.)

[3] [Birth Rate by Country, (n.d.).](http://paperpile.com/b/Zxqofu/yx2hK) <https://www.macrotrends.net/global-metrics/countries/ranking/birth-rate> [(accessed February 15, 2025).](http://paperpile.com/b/Zxqofu/yx2hK)

[4] [Death Rate by Country, (n.d.).](http://paperpile.com/b/Zxqofu/UrAiA) <https://www.macrotrends.net/global-metrics/countries/ranking/death-rate> [(accessed February 15, 2025).](http://paperpile.com/b/Zxqofu/UrAiA)

[5] [Population Pyramids of the World from 1950 to 2100, PopulationPyramid.net (n.d.).](http://paperpile.com/b/Zxqofu/fy0g) <https://www.populationpyramid.net/australianew-zealand/2021/> [(accessed October 18, 2025).](http://paperpile.com/b/Zxqofu/fy0g)

[6] [Government of Canada, S. Canada, Profile table, Census Profile, 2021 Census of Population - Canada [Country], (2022).](http://paperpile.com/b/Zxqofu/BYJW6) <https://www12.statcan.gc.ca/census-recensement/2021/dp-pd/prof/details/page.cfm?Lang=E&DGUIDList=2021A000011124&GENDERList=1&STATISTICList=1&HEADERList=0&SearchText=Canada> [(accessed February 11, 2025).](http://paperpile.com/b/Zxqofu/BYJW6)

[7] [Population Pyramids of the World from 1950 to 2100, PopulationPyramid.net (n.d.).](http://paperpile.com/b/Zxqofu/O1Id) <https://www.populationpyramid.net/france/2021/> [(accessed October 18, 2025).](http://paperpile.com/b/Zxqofu/O1Id)

[8] [Population by age groups (from 2011), Federal Statistical Office (n.d.).](http://paperpile.com/b/Zxqofu/tuB85) <https://www.destatis.de/EN/Themes/Society-Environment/Population/Current-Population/Tables/liste-agegroups.html> [(accessed February 15, 2025).](http://paperpile.com/b/Zxqofu/tuB85)

[9] [Population Pyramids of the World from 1950 to 2100, PopulationPyramid.net (n.d.).](http://paperpile.com/b/Zxqofu/MQQjZ) <https://www.populationpyramid.net/ireland/> [(accessed February 11, 2025).](http://paperpile.com/b/Zxqofu/MQQjZ)

[10] [country or area, T.U.-U.R.-. Date, A. (in, 7. Population by age, sex and urban/rural residence: latest available year, 2011 - 2020 Population selon l’âge, le sexe et la résidence, urbaine/rurale : dernière année disponible, 2011 - 2020, (n.d.).](http://paperpile.com/b/Zxqofu/QXC2A) <https://unstats.un.org/unsd/demographic-social/products/dyb/documents/DYB2020/table07.pdf> [(accessed February 11, 2025).](http://paperpile.com/b/Zxqofu/QXC2A)

[11] [Italy: population by age group 2024, Statista (n.d.).](http://paperpile.com/b/Zxqofu/abWC5) <https://www.statista.com/statistics/789270/population-in-italy-by-age-group/> [(accessed February 11, 2025).](http://paperpile.com/b/Zxqofu/abWC5)

[12] [Population Census 2020 Population Census Basic Complete Tabulation on Population and Households 2020Oct, Portal Site of Official Statistics of Japan (n.d.).](http://paperpile.com/b/Zxqofu/jOqo0) <https://www.e-stat.go.jp/en/stat-search/files?page=1&layout=datalist&toukei=00200521&tstat=000001136464&cycle=0&year=20200&month=24101210&tclass1=000001136466> [(accessed February 11, 2025).](http://paperpile.com/b/Zxqofu/jOqo0)

[13] [Population Pyramids of the World from 1950 to 2100, PopulationPyramid.net (n.d.).](http://paperpile.com/b/Zxqofu/yVLx2) <https://www.populationpyramid.net/spain/2023/> [(accessed February 11, 2025).](http://paperpile.com/b/Zxqofu/yVLx2)

[14] [Population by age and sex. Year 1860 - 2023, Statistikdatabasen (n.d.).](http://paperpile.com/b/Zxqofu/wXmAR) <https://www.statistikdatabasen.scb.se/pxweb/en/ssd/START__BE__BE0101__BE0101A/BefolkningR1860N/table/tableViewLayout1/> [(accessed February 11, 2025).](http://paperpile.com/b/Zxqofu/wXmAR)

[15] [S. Netherlands, Population pyramid, Statistics Netherlands (n.d.).](http://paperpile.com/b/Zxqofu/3L2HG) <https://www.cbs.nl/en-gb/visualisations/dashboard-population/population-pyramid> [(accessed February 11, 2025).](http://paperpile.com/b/Zxqofu/3L2HG)

[16] [UK population pyramid interactive, (2020).](http://paperpile.com/b/Zxqofu/oYbiN) <https://www.ons.gov.uk/peoplepopulationandcommunity/populationandmigration/populationestimates/articles/ukpopulationpyramidinteractive/2020-01-08> [(accessed February 11, 2025).](http://paperpile.com/b/Zxqofu/oYbiN)

[17] [Age groups, (2023).](http://paperpile.com/b/Zxqofu/fOMh3) <https://www.ethnicity-facts-figures.service.gov.uk/uk-population-by-ethnicity/demographics/age-groups/latest> [(accessed February 11, 2025).](http://paperpile.com/b/Zxqofu/fOMh3)

[18] [US Census Bureau, America’s Families and Living Arrangements: 2019, (2019).](http://paperpile.com/b/Zxqofu/KJbLk) <https://www.census.gov/data/tables/2019/demo/families/cps-2019.html> [(accessed February 11, 2025).](http://paperpile.com/b/Zxqofu/KJbLk)

[19] [Household size, (n.d.).](http://paperpile.com/b/Zxqofu/5guJn) <https://profile.id.com.au/australia/household-size?WebID=10&BMID=50.> [(accessed February 10, 2025).](http://paperpile.com/b/Zxqofu/5guJn)

[20] [Household size in France 2015, Statista (n.d.).](http://paperpile.com/b/Zxqofu/HUNlY) <https://www.statista.com/statistics/512974/number-households-by-size-france/> [(accessed February 11, 2025).](http://paperpile.com/b/Zxqofu/HUNlY)

[21] [GENESIS-Online, (n.d.).](http://paperpile.com/b/Zxqofu/yFRcV) <https://www-genesis.destatis.de/genesis/online> [(accessed February 11, 2025).](http://paperpile.com/b/Zxqofu/yFRcV)

[22] [UNdata, (n.d.).](http://paperpile.com/b/Zxqofu/fX7mP) <https://data.un.org/Data.aspx?d=POP&f=tableCode:50> [(accessed February 11, 2025).](http://paperpile.com/b/Zxqofu/fX7mP)

[23] [United Nations Statistics Division, Demographic and Social Statistics, (n.d.).](http://paperpile.com/b/Zxqofu/Tem4n) <https://unstats.un.org/unsd/demographic-social/products/dyb/.> [(accessed February 11, 2025).](http://paperpile.com/b/Zxqofu/Tem4n)

[24] [OECD, Aspects of daily life - Household : Households size, (n.d.).](http://paperpile.com/b/Zxqofu/anpaa) <http://dati.istat.it/Index.aspx?QueryId=18306&lang=en> [(accessed February 11, 2025).](http://paperpile.com/b/Zxqofu/anpaa)

[25] [UNdata, (n.d.).](http://paperpile.com/b/Zxqofu/NnSos) <http://data.un.org/Data.aspx?d=POP&f=tableCode:50> [(accessed February 10, 2025).](http://paperpile.com/b/Zxqofu/NnSos)

[26] [Number and percentage of households by region, type of housing and size of household. Year 2012 - 2023, Statistikdatabasen (n.d.).](http://paperpile.com/b/Zxqofu/pWvNg) <https://www.statistikdatabasen.scb.se/pxweb/en/ssd/START__HE__HE0111__HE0111A/HushallT26/> [(accessed February 11, 2025).](http://paperpile.com/b/Zxqofu/pWvNg)

[27] [(n.d.).](http://paperpile.com/b/Zxqofu/Bwbby) <https://population.un.org/Household/index.html#/countries/528> [(accessed February 11, 2025).](http://paperpile.com/b/Zxqofu/Bwbby)

[28] [Families and households, (2024).](http://paperpile.com/b/Zxqofu/VsFs1) <https://www.ons.gov.uk/peoplepopulationandcommunity/birthsdeathsandmarriages/families/datasets/familiesandhouseholdsfamiliesandhouseholds> [(accessed February 11, 2025).](http://paperpile.com/b/Zxqofu/VsFs1)

[29] [Government of Canada, S. Canada, Housing Indicators (5), Tenure Including Presence of Mortgage Payments and Subsidized Housing (7), Age of Primary Household Maintainer (9), Household Type Including Census Family Structure (9) and Household Size (8) for Owner and Tenant Households With Household Total Income Greater Than Zero in Non-farm, Non-reserve Private Dwellings of Canada, Provinces and Territories, Census Divisions and Census Subdivisions, 2016 Census - 25% Sample Data, (2017).](http://paperpile.com/b/Zxqofu/zEZWh) <https://www12.statcan.gc.ca/census-recensement/2016/dp-pd/dt-td/Rp-eng.cfm?TABID=2&LANG=E&A=R&APATH=3&DETAIL=0&DIM=0&FL=A&FREE=0&GC=01&GL=-1&GID=1257309&GK=1&GRP=1&O=D&PID=110575&PRID=10&PTYPE=109445&S=0&SHOWALL=0&SUB=0&Temporal=2017&THEME=121&VID=0&VNAMEE=&VNAMEF=&D1=4&D2=0&D3=1&D4=0&D5=0&D6=0> [(accessed February 11, 2025).](http://paperpile.com/b/Zxqofu/zEZWh)

[30] [UNdata, (n.d.).](http://paperpile.com/b/Zxqofu/orEUJ) <https://data.un.org/Data.aspx?d=POP&f=tableCode%3A326> [(accessed February 11, 2025).](http://paperpile.com/b/Zxqofu/orEUJ)

[31] [(n.d.).](http://paperpile.com/b/Zxqofu/IXIf5) [https://www.cbs.gov.il/en/publications/Pages/2022/Households–Economic-Characteristics-and-Housing-Density-Based-on-Labour-Force-Survey-2020.aspx](https://www.cbs.gov.il/en/publications/Pages/2022/Households%E2%80%93Economic-Characteristics-and-Housing-Density-Based-on-Labour-Force-Survey-2020.aspx) [(accessed February 11, 2025).](http://paperpile.com/b/Zxqofu/IXIf5)

[32] [(n.d.).](http://paperpile.com/b/Zxqofu/tVZKw) <http://data.un.org/Data.aspx?d=POP&f=tableCode:50.> [(accessed February 11, 2025).](http://paperpile.com/b/Zxqofu/tVZKw)

[33] [(n.d.).](http://paperpile.com/b/Zxqofu/Qxi2j) <https://data.un.org/Data.aspx?q=household+size&d=POP&f=tableCode:50.> [(accessed February 11, 2025).](http://paperpile.com/b/Zxqofu/Qxi2j)

[34] [Number of households by household size and age of household reference person (HRP), English regions and UK constituent countries, 2019, Office for National Statistics (2019).](http://paperpile.com/b/Zxqofu/mfOz2)

[35] [Schools, Australian Bureau of Statistics (2024).](http://paperpile.com/b/Zxqofu/DLUMp) <https://www.abs.gov.au/statistics/people/education/schools/latest-release#data-download> [(accessed February 10, 2025).](http://paperpile.com/b/Zxqofu/DLUMp)

[36] [Government of Canada, S. Canada, Postsecondary enrolments, by registration status, institution type, status of student in Canada and gender, (2024).](http://paperpile.com/b/Zxqofu/m1DVP) <https://www150.statcan.gc.ca/t1/tbl1/en/tv.action?pid=3710001801&pickMembers%5B0%5D=2.3&pickMembers%5B1%5D=5.1&pickMembers%5B2%5D=7.1&pickMembers%5B3%5D=4.1&pickMembers%5B4%5D=6.1&cubeTimeFrame.startYear=2019+%2F+2020&cubeTimeFrame.endYear=2019+%2F+2020&referencePeriods=20190101%2C20190101> [(accessed February 11, 2025).](http://paperpile.com/b/Zxqofu/m1DVP)

[37] [Council of Ministers of Education, Canada, CMEC (n.d.).](http://paperpile.com/b/Zxqofu/VykEx) <https://www.cmec.ca/299/education-in-canada-an-overview/index.html> [(accessed February 11, 2025).](http://paperpile.com/b/Zxqofu/VykEx)

[38] [Government of Canada, S. Canada, Number of students enrolled in kindergarten, (2021).](http://paperpile.com/b/Zxqofu/5JTN7) <https://www150.statcan.gc.ca/t1/tbl1/en/tv.action?pid=4210001401> [(accessed February 11, 2025).](http://paperpile.com/b/Zxqofu/5JTN7)

[39] [Schools, pupils and their characteristics, (n.d.).](http://paperpile.com/b/Zxqofu/nsr8h) <https://explore-education-statistics.service.gov.uk/find-statistics/school-pupils-and-their-characteristics> [(accessed February 11, 2025).](http://paperpile.com/b/Zxqofu/nsr8h)

[40] [D. Lawlor, S.P. Researcher, S. Burke, S.P. Researcher, EDUCATION IN IRELAND, (n.d.).](http://paperpile.com/b/Zxqofu/K2CN1) <https://data.oireachtas.ie/ie/oireachtas/libraryResearch/2020/2020-04-03_l-rs-infographic-education-in-ireland-a-statistical-snapshot_en.pdf> [(accessed February 11, 2025).](http://paperpile.com/b/Zxqofu/K2CN1)

[41] [Education- Statistical Abstract of Israel 2020 - No.71, (n.d.).](http://paperpile.com/b/Zxqofu/ZnzyV) <https://www.cbs.gov.il/en/publications/Pages/2020/Education-Statistical-Abstract-of-Israel-2020-No-71.aspx> [(accessed February 11, 2025).](http://paperpile.com/b/Zxqofu/ZnzyV)

[42] [Study in Israel: Education in Israel, Https://www.educations.com (2025).](http://paperpile.com/b/Zxqofu/0bos3) <https://www.educations.com/study-guides/asia/study-in-israel/education-system-17589> [(accessed February 11, 2025).](http://paperpile.com/b/Zxqofu/0bos3)

[43] [I. Israel, You Can Open a Daycare Center in Just Two Minutes - Israel News - Haaretz, n.d.](http://paperpile.com/b/Zxqofu/lVk1S)

[44] [Education and training, (2024).](http://paperpile.com/b/Zxqofu/HeoTJ) <https://www.istat.it/en/education-and-training?data-and-indicators> [(accessed February 11, 2025).](http://paperpile.com/b/Zxqofu/HeoTJ)

[45] [Italy: number of public universities by region 2020, Statista (n.d.).](http://paperpile.com/b/Zxqofu/NNFmA) <https://www.statista.com/statistics/728414/number-of-public-universities-by-region-italy/> [(accessed February 11, 2025).](http://paperpile.com/b/Zxqofu/NNFmA)

[46] [MEXT releases the results of the FY2021 School Basic Survey, NIC-Japan, National Information Center for Academic Recognition Japan (2022).](http://paperpile.com/b/Zxqofu/xW3kD) <https://www.nicjp.niad.ac.jp/en/news/schoolbasicsurvey2021.html> [(accessed February 11, 2025).](http://paperpile.com/b/Zxqofu/xW3kD)

[47] [(n.d.).](http://paperpile.com/b/Zxqofu/7H6xu) <https://www.educacionfpydeportes.gob.es/dam/jcr:4d443eb4-40e5-4be4-b739-dd396aa5852f/datos-y-cifras-2021-2022-english.pdf> [(accessed February 11, 2025).](http://paperpile.com/b/Zxqofu/7H6xu)

[48] [Short-cycle higher education, in: The International Encyclopedia of Higher Education Systems and Institutions, Springer Netherlands, Dordrecht, 2020: pp. 2534–2534. https://doi.org/](http://paperpile.com/b/Zxqofu/5E6Ma)[10.1007/978-94-017-8905-9_300683.](http://dx.doi.org/10.1007/978-94-017-8905-9_300683.)

[49] [Children and kindergartens, (n.d.).](http://paperpile.com/b/Zxqofu/Yf5wF) <https://www.udir.no/in-english/the-education-mirror-2022/kindergarten/children-and-kindergartens/.> [(accessed February 11, 2025).](http://paperpile.com/b/Zxqofu/Yf5wF)

[50] [Facts about education in Norway 2023, SSB (2023).](http://paperpile.com/b/Zxqofu/GfWw5) <https://www.ssb.no/en/utdanning/utdanningsniva/artikler/facts-about-education-in-norway-2023> [(accessed February 11, 2025).](http://paperpile.com/b/Zxqofu/GfWw5)

[51] [CBS Statline, (n.d.).](http://paperpile.com/b/Zxqofu/hebJS) <https://opendata.cbs.nl/#/CBS/en/dataset/03753eng/table?searchKeywords=school> [(accessed February 11, 2025).](http://paperpile.com/b/Zxqofu/hebJS)

[52] [Digest of Education Statistics, 2019, (n.d.).](http://paperpile.com/b/Zxqofu/mUeP7) <https://nces.ed.gov/pubsearch/pubsinfo.asp?pubid=2021009> [(accessed February 10, 2025).](http://paperpile.com/b/Zxqofu/mUeP7)

[53] [Counts of Australian Businesses, including Entries and Exits, Australian Bureau of Statistics (2024).](http://paperpile.com/b/Zxqofu/yoeNc) <https://www.abs.gov.au/statistics/economy/business-indicators/counts-australian-businesses-including-entries-and-exits/latest-release#data-download> [(accessed February 10, 2025).](http://paperpile.com/b/Zxqofu/yoeNc)

[54] [Government of Canada, S. Canada, Employment for all employees by enterprise size, annual, (2024).](http://paperpile.com/b/Zxqofu/GYul8) <https://www150.statcan.gc.ca/t1/tbl1/en/tv.action?pid=1410021501&pickMembers%5B0%5D=1.1&pickMembers%5B1%5D=3.2&cubeTimeFrame.startYear=2021&cubeTimeFrame.endYear=2021&referencePeriods=20210101%2C20210101> [(accessed February 11, 2025).](http://paperpile.com/b/Zxqofu/GYul8)

[55] [Number of companies in France 2020, by number of employees, Statista (n.d.).](http://paperpile.com/b/Zxqofu/fqZ5v) <https://www.statista.com/statistics/502717/number-of-enterprises-france-by-number-employees/> [(accessed February 11, 2025).](http://paperpile.com/b/Zxqofu/fqZ5v)

[56] [Ireland Industry Breakdown: Top Industries & Companies, (n.d.).](http://paperpile.com/b/Zxqofu/h7jn1) <https://www.hithorizons.com/eu/analyses/country-statistics/ireland#:~:text=The%20number%20of%20companies%20in%20Ireland%20is%20353%2C193%2C,registered%20companies%20is%20United%20Kingdom%20with%206%2C517%2C611%20companies.> [(accessed February 11, 2025).](http://paperpile.com/b/Zxqofu/h7jn1)

[57] [SMEs in Ireland 2023, by size, Statista (n.d.).](http://paperpile.com/b/Zxqofu/uOwZD) <https://www.statista.com/statistics/879089/number-of-smes-in-ireland/> [(accessed February 11, 2025).](http://paperpile.com/b/Zxqofu/uOwZD)

[58] [Publications, OECD (n.d.).](http://paperpile.com/b/Zxqofu/eIOpc) <https://www.oecd.org/en/publications.html#:~:text=Micro%2Denterprises%20(1%2D9,of%20businesses%20compared%20with%20an> [(accessed February 11, 2025).](http://paperpile.com/b/Zxqofu/eIOpc)

[59] [Economic Census for Business Frame 2019 Economic Census for Business Frame Privately Owned Tabulation of Newly Grasped Establishments Results for Japan, Portal Site of Official Statistics of Japan (n.d.).](http://paperpile.com/b/Zxqofu/uE1JO) <https://www.e-stat.go.jp/en/stat-search/files?page=1&layout=datalist&toukei=00200552&tstat=000001137226&cycle=0&tclass1=000001137228&tclass2=000001137230&tclass3=000001147806&tclass4val=0> [(accessed February 11, 2025).](http://paperpile.com/b/Zxqofu/uE1JO)

[60] [Employees by business size, OECD (n.d.).](http://paperpile.com/b/Zxqofu/vDBMt) <https://data.oecd.org/entrepreneur/employees-by-business-size.htm> [(accessed February 11, 2025).](http://paperpile.com/b/Zxqofu/vDBMt)

[61] [(n.d.).](http://paperpile.com/b/Zxqofu/AsAYk) <https://www.scb.se/en/finding-statistics/statistics-by-subject-area/business-activities/structure-of-the-business-sector/structural-business-statistics/> [(accessed February 11, 2025).](http://paperpile.com/b/Zxqofu/AsAYk)

[62] [Distribution of total enterprises in the Netherlands 2022, by size, Statista (n.d.).](http://paperpile.com/b/Zxqofu/uI11U) <https://www.statista.com/statistics/870174/distribution-of-total-enterprises-in-the-netherlands-by-size/> [(accessed February 11, 2025).](http://paperpile.com/b/Zxqofu/uI11U)

[63] [No dataset selected - Nomis - Official Census and Labour Market Statistics, (n.d.).](http://paperpile.com/b/Zxqofu/4H3dB) <https://www.nomisweb.co.uk/query/construct/components/stdListComponent.asp?menuopt=12&subcomp=100> [(accessed February 11, 2025).](http://paperpile.com/b/Zxqofu/4H3dB)

[64] [U.S. Census Bureau, Explore Census Data, (n.d.).](http://paperpile.com/b/Zxqofu/DbPL1) <https://data.census.gov/all?q=United%20States%20Census%20Bureau> [(accessed February 15, 2025).](http://paperpile.com/b/Zxqofu/DbPL1)

[65] [[No title], (n.d.).](http://paperpile.com/b/Zxqofu/C9O70) [https://data-explorer.oecd.org/vis?tenant=archive&df[ds]=DisseminateArchiveDMZ&df[id]=DF_EAG_ENRL_RATE_AGE&df[ag]=OECD.](https://data-explorer.oecd.org/vis?tenant=archive&df%5Bds%5D=DisseminateArchiveDMZ&df%5Bid%5D=DF_EAG_ENRL_RATE_AGE&df%5Bag%5D=OECD.) [(accessed February 15, 2025).](http://paperpile.com/b/Zxqofu/C9O70)

[66] [Government of Canada, S. Canada, Labour force characteristics by gender and detailed age group, monthly, unadjusted for seasonality (x 1,000), (2025).](http://paperpile.com/b/Zxqofu/b7ZmI) <https://www150.statcan.gc.ca/t1/tbl1/en/tv.action?pid=1410001701> [(accessed February 11, 2025).](http://paperpile.com/b/Zxqofu/b7ZmI)

[67] [Government of Canada, S. Canada, Age (in single years), average age and median age and gender: Canada, provinces and territories and economic regions, (2022).](http://paperpile.com/b/Zxqofu/RZWgJ) <https://www150.statcan.gc.ca/t1/tbl1/en/tv.action?pid=9810002601> [(accessed February 11, 2025).](http://paperpile.com/b/Zxqofu/RZWgJ)

[68] [Labour Market and Education, (n.d.).](http://paperpile.com/b/Zxqofu/olBhA) <https://www.instat.gov.al/en/themes/labour-market-and-education/> [(accessed February 11, 2025).](http://paperpile.com/b/Zxqofu/olBhA)

[69] [Employment rate by age group, OECD (n.d.).](http://paperpile.com/b/Zxqofu/2vmpv) <https://data.oecd.org/emp/employment-rate-by-age-group.htm> [(accessed February 11, 2025).](http://paperpile.com/b/Zxqofu/2vmpv)

[70] [Statistics Bureau, Ministry of Internal Affairs, Communications, Statistics Bureau Home Page/Labour Force Survey/Historical data, (n.d.).](http://paperpile.com/b/Zxqofu/FwEUS) <https://www.stat.go.jp/english/data/roudou/lngindex.html> [(accessed February 11, 2025).](http://paperpile.com/b/Zxqofu/FwEUS)

[71] [Government of Canada, S. Canada, Participation rate in education, population aged 15 to 29, by age and type of institution attended, (2024).](http://paperpile.com/b/Zxqofu/NS0jN) <https://www150.statcan.gc.ca/t1/tbl1/en/tv.action?pid=3710010101&cubeTimeFrame.startYear=2020+%2F+2021&cubeTimeFrame.endYear=2020+%2F+2021&referencePeriods=20200101%2C20200101> [(accessed February 11, 2025).](http://paperpile.com/b/Zxqofu/NS0jN)

[72] [[No title], (n.d.).](http://paperpile.com/b/Zxqofu/ejbqW) [https://data-explorer.oecd.org/vis?tenant=archive&df[ds]=DisseminateArchiveDMZ&df[id]=DF_EAG_ENRL_RATE_AGE&df[ag]=OECD](https://data-explorer.oecd.org/vis?tenant=archive&df%5Bds%5D=DisseminateArchiveDMZ&df%5Bid%5D=DF_EAG_ENRL_RATE_AGE&df%5Bag%5D=OECD) [(accessed February 11, 2025).](http://paperpile.com/b/Zxqofu/ejbqW)

[73] [Statistics Bureau, Ministry of Internal Affairs, Communications, Statistics Bureau Home Page/JAPAN STATISTICAL YEARBOOK 2015 - Chapter 22 Education, (n.d.).](http://paperpile.com/b/Zxqofu/PbmNq) <https://www.stat.go.jp/english/data/nenkan/back64/1431-22.html> [(accessed February 11, 2025).](http://paperpile.com/b/Zxqofu/PbmNq)
